# Growth Inhibition and Apoptosis Induction by (+)-Cyanidan-3-ol in Hepatocellular Carcinoma

**DOI:** 10.1371/journal.pone.0068710

**Published:** 2013-07-24

**Authors:** Jitender Monga, Saurabh Pandit, Rajinder Singh Chauhan, Chetan Singh Chauhan, Shailender Singh Chauhan, Manu Sharma

**Affiliations:** 1 Department of Pharmacy, Jaypee University of Information Technology, Waknaghat, Himachal Pradesh, India; 2 Department of Biotechnology & Bioinformatics, Jaypee University of Information Technology, Waknaghat, Himachal Pradesh, India; 3 Department of Pharmacy, Bhupal Noble College of Pharmacy, Udaipur, Rajasthan, India; 4 Department of Gastroenterology, Post Graduate Institute of Medical Research, Chandigarh, India; Wayne State University, United States of America

## Abstract

The objective of this study was to evaluate the cytotoxicity of (+)-cyanidan-3-ol (CD-3) in human hepatocellular carcinoma cell line (HepG2) and chemopreventive potential against hepatocellular carcinoma (HCC) in Balb/c mice. The HepG2 cell line was treated with CD-3 at various concentrations and the proliferation of the HepG2 cells was measure by 3-[4,5-dimethylthiazol-2-yl]-2,5-diphenyl-tetrazolium bromide (MTT), sulforhodamine B (SRB) and lactate dehydrogenase (LDH) assays. Cell apoptosis was detected by Hoechst 33258 (HO), Acridine orange/ethylene dibromide (AO/EB) staining, DNA fragmentation analysis and the apoptosis rate was detected by flow cytometry. The HCC tumor model was established in mice by injecting *N*-nitrosodiethylamine/carbon tetrachloride (NDEA/CCl_4_) and the effect of CD-3 on tumor growth *in-vivo* was studied. The levels of liver injury markers, tumor markers, and oxidative stress were measured. The expression levels of apoptosis-related genes in *in-vitro* and *in vivo* models were determined by RT-PCR and ELISA. The CD-3 induced cell death was considered to be apoptotic by observing the typical apoptotic morphological changes under fluorescent microscopy and DNA fragmentation analysis. Annexin V/PI assay demonstrated that apoptosis increased with increase in the concentration of CD-3. The expression levels of apoptosis-related genes that belong to bcl-2 and caspase family were increased and AP-1 and NF-κB activities were significantly suppressed by CD-3. Immunohistochemistry data revealed less localization of p53, p65 and c-jun in CD-3 treated tumors as compared to localization in NDEA/CCl_4_ treated tumors. Taken together, our data demonstrated that CD-3 could significantly inhibit the proliferation of HepG2 cells *in-vitro* and suppress HCC tumor growth *in-vivo* by apoptosis induction.

## Introduction

Hepatocellular carcinoma (HCC) is one of the most frequent tumors representing the fifth commonest malignancy worldwide and the third cause of mortality from cancer. The regions of high incidence are Eastern and South-Eastern Asia, Middle and Western Africa, Southern Europe as well as South America and kill an astounding number of people every year [1]. Unfortunately, the overall response rate of liver cancer treatment is unsatisfactory mainly due to late diagnosis and poor treatment efficacy, especially resistance to chemotherapeutic drugs and metastasis to other organs [2]. Thus, the development of new and effective therapeutic strategies for liver cancer has a greater need and importance.

**Figure 1:**
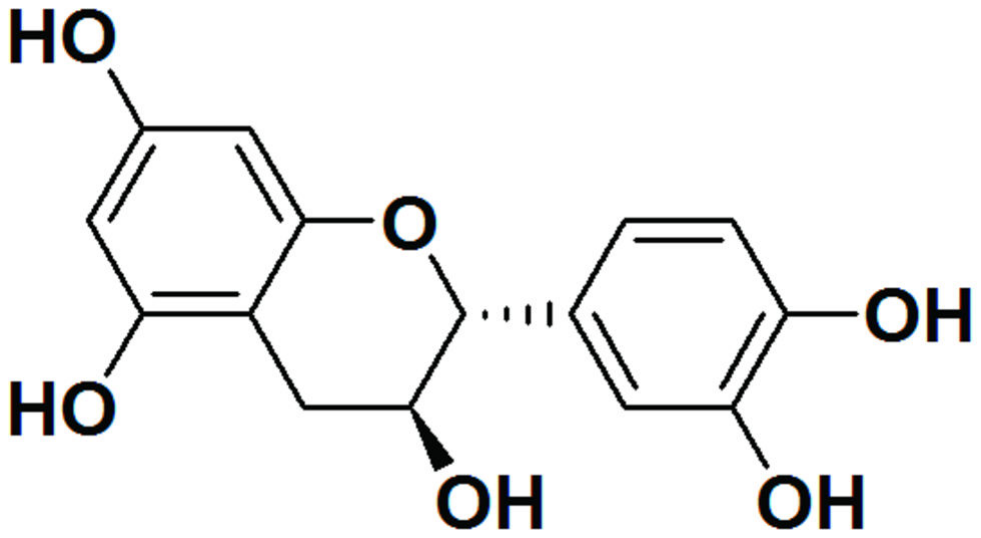
Chemical structure of (+)-cyanidan-3-ol.

In recent years, the number of natural products has acquired a lot of attention because of their ability to provide prevention and therapeutic efficacy against number of cancers [3]. Out of number of different classes of natural products, flavonoids represent a diverse group of low molecular weight polyphenolic compounds that are widely distributed in nature and renewed interest has been observed in recent years in the novel and multiple activities of flavonoids [4]. *Acacia* *catechu* Willd. (Fabaceae) also known as ‘Khadira’ has been extensively used as traditional medicine in India/Asia over a long period of time and a rich source of number of polyphenolic compounds. Previously, we have reported chemopreventive effect of aqueous extract of *Acacia* *catechu* in skin and mammary cancer rodent models [5,6]. (+)-Cyanidan-3-ol (CD-3) [3',4',5,7-tetrahydroxyflavan-3-ol] (Figure 1) is the most abundant polyphenolic flavonoid in the *Acacia* *catechu* heartwood and studies of the biological effects of CD-3 in cell culture and *in vivo* models indicated that this compound can inhibit lipid peroxidation [7]. CD-3 is claimed to be effective in treating carbon tetrachloride induced liver damage [8] and also reported to inhibit angiogenesis *in-vivo* [9]. However, there is no report on the effect of CD-3 on hepatocellular carcinoma (HCC). In this paper, we report the chemopreventive and therapeutic efficacy of CD-3 against hepatocellular carcinoma by using both *in-vitro* and *in vivo* systems. Further, the underlying cellular andmolecular mechanisms of CD-3 actions were also evaluated. Our data provided investigational evidence to carry the potential development of CD-3 as an efficient and safe candidate for the prevention and/or therapy of liver cancer.

## Materials and Methods

### Antibodies and Reagents

All the chemicals used in the study (analytical grade) were obtained from Sigma Chemical Co. (St. Louis, MO, USA), Merck (Mumbai, India), and Sigma Himedia Laboratories (Mumbai, India). Antibodies against p53, p65, c-jun, bcl-2, bax and caspase-3 were obtained from Santa Cruz Biotechnology, Santa Cruz, CA (USA). Annexin V-FITC apoptosis detection kit was obtained from EMD biosciences (Calbiochem, Inc, USA).

### Extraction of *Acacia* *catechu* heartwood and isolation of (+)-cyanidan-3-ol

*Acacia* *catechu* heartwood was collected from Hamirpur, Himachal Pradesh, India during the month of September, 2011. The plant material was taxonomically identified and authenticated by Dr. Sunil Dutta, Scientist, National Medicinal Plant Board, Ayush, New Delhi, India. A voucher specimen (AC-2011) was deposited in the herbarium at Pharmacy Department, Jaypee University of Information Technology, Waknaghat, Himachal Pradesh.

A total of one kg of the dried powder of *A* *. catechu* heartwood was put in an aluminium pot with ten litre of water and boiled for 5 h and was then allowed to stand for 24 h. The extract was decanted and filtered through a fine muslin cloth to remove suspended materials. The filtrate was evaporated and the residue obtained was air dried to obtain a solid mass (212 g), with 21.2%, yield. Solid mass (150 g) was added to five litre stainless steel beaker containing one litre distilled water. It was boiled with constant stirring for complete dissolution and then filtered. It was then evaporated to 500 ml and allowed to stand for 24 h. The aqueous filtrate was rejected, and the residue was dissolve in ethanol and filtered. The ethanolic solution was evaporated to dryness and the residue was dissolved in 500 ml hot water and was allowed to stand for 24 h. The precipitate was filtered and dried in air and the process of re-crystallization from water was repeated three times (m.p. 95–6 °C, yield 37.5 g, 25%). The white crystals of (+)-cyanidan-3-ol was isolated from *Acacia* *catechu* heartwood and characterized by spectral analysis. The purity of (+)-cyanidan-3-ol was confirmed by HPLC (Figure S1).

IR (ν_max_, KBr, cm^-1^): 3501.59, 3456.74, 3412.9, 3176.44 (br, O–H stretch), 2931.38 (C–H stretch), 1624.88, 1521.69 (C=C stretch), 1183.59, 1143.77(C–O stretch). ^1^H NMR (CDCl_3_, δ ppm): 9.0370 (5H, br, 5 OH groups), 6.7355-6.7403 (1H, d, *J*= 1.92, C-2′-H), 6.6757-6.6958 (1H, d, *J*= 8.04, C-5′-H), 6.5774-6.6027 (1H, dd, *J*= 8.16, 1.96, C-6′-H), 5.8837-5.8894 (1H, d, *J*= 2.28, C-8-H), 5.6976-5.7032 (1H, d, *J*= 2.24, C-6-H), 4.4670-4.4856 (1H, d, *J*= 7.44, C-2-H), 3.7993-3.8514 (1H, m, C-3-H), 2.6536-2.7070 (2H, dd, *J*= 16.04, 5.36, C-4-H)^13^. C NMR (CDCl_3_, δ ppm): 156.33 (C-7), 156.05 (C-5), 155.23 (C-9), 144.72 (C-3′), 144.60 (C-4′), 130.50 (C-1′), 118.27 (C-2′), 114.92 (C-5′), 114.31 (C-6′), 98.96 (C-10), 95.04 (C-8), 93.80 (C-6), 80.98 (C-2), 66.39 (C-3), 27.67 (C-4). ESI-MS (*m/z*): 289(M^+^-1).

### Cell culture and treatment

Human hepatocarcinoma cell line (HepG2) was obtained from National Centre for Cancer Science (NCCS), Pune and grown as a monolayer in DMEM supplemented with 10% FBS (Fetal Bovine Serum), 100 µg/ml streptomycin and 100 units/ml penicillin. Cells were incubated at 37 °C in an atmosphere of 5% CO_2_. Cells were grown to 85% confluence and treated with CD-3 for 48 h. Human lymphocytes were isolated from peripheral blood [10]. Lymphocytes were suspended in complete RPMI 1640 medium supplemented with 10% FBS, 5 g/ml phytohemagglutinin (PHA), and maintained at 37 °C in a 5% CO_2_ humidified incubator. Stock solutions and dilutions of CD-3 were prepared in culture medium. For 96 well plates, cell were seeded at approximately 1.5 × 10^4^ cells per well. For 24 well plates, cells were seeded at approximately 4 × 10^4^ cells per well.

### MTT assay

The cell viability of HepG2 cells was assessed by the MTT colorimetric assay [11]. Briefly, the adherent HepG2 cells were incubated in 96-well microtiter plates for 48 h at 37 °C in a 5% CO_2_ incubator. Following the addition of test compound, the plates were incubated for an additional 48 h. Control wells contained medium alone and three replicate wells were used at each point in the experiments. After 48 h incubation, MTT solution (5 mg/ml in phosphate-buffered saline) was added and incubated for another 4 h. The resulting MTT/formazan product was dissolved by 100 µl of isopropanol and the plates were gently shaken to solubilise the formed formazan. The amount of formazan was determined by measuring the absorbance (OD) at 570 nm using a Bio-Rad 550 enzyme-linked immunosorbent assay (ELISA) microplate reader.

### Sulforhodamine B (SRB) assay

Growth inhibition was determined using the SRB assay which estimates cell number indirectly by measuring total basic amino acids [12]. Briefly, the cells were incubated in 96-well microtiter plates for 48 h. Following the addition of test compound, the plates were incubated at 37 °C for an additional 48 h in a 5% CO_2_ incubator. The culture medium was then discarded and the cells were fixed *in situ* by the gentle addition of 100 µl of cold 10% (w/v) trichloroacetic acid and incubated for 60 min at 4 °C. The supernatant was discarded and the plates were washed five times with tap water and air dried. SRB solution (100 µl) at 0.4% (w/v) in 1% acetic acid was added and plates were incubated for 20 min at room temperature. After staining, unbound dye was removed by washing five times with 1% acetic acid and the plates were air dried. Bound stain was subsequently solubilised with 10 mM Tris (pH 10.5) and the absorbance was read at 515 nm on a Bio-Rad 550 ELISA microplate reader.

### Lactate dehydrogenase (LDH) leakage assay

In LDH assay, leakage of the cytoplasmic located enzyme LDH into the extracellular medium is measured [13]. LDH activity was measured in both the supernatants and the cell lysate fractions using Cyto-Tox 96, a non-radioactive cytotoxicity assay kit (Promega, WI, USA), in accordance with the manufacturer’s instruction and the intensity of color is proportional to LDH activity. LDH release was calculated by measuring the absorbance at 490 nm using ELISA microplate reader.

### Morphological changes of cells

Analysis of cell morphological changes was evaluated for 48 h in the absence or presence of CD-3 at concentrations of 10, 20, 30, 40 and 50 µg/ml in a 24-well plate. At the end of the incubation period, cells were observed under phase contrast inverted microscope at 200× magnification.

### Hoechst assay

Differential staining with specific fluorochromes can be used to distinguish cells undergoing apoptosis from viable and necrotic cells. Analysis of changes in cell morphology was evaluated by Hoechst fluorescence staining [14]. The cells were cultured in 24-well plates to 85% confluence and then treated with the compound at concentrations of 25 and 35 µg/ml for 48 h at 37 °C. The cells were washed in PBS and fixed with 4% formaldehyde in PBS for 15 min and stained with 10 µg/ml Hoechst 33258 (HO; Sigma, St. Louis, MO, USA) at room temperature for 10 min in PBS. The apoptosis was classified by morphology and color of the cells, and quantified. Finally, after the cells were washed with PBS, morphological changes were observed under a fluorescence microscope (Nikon Eclipse Ti, Japan).

### Acridine orange/ ethidium bromide (AO/EB) fluorescence staining

Analysis of changes in cell morphology was evaluated using AO/EB fluorescence staining [15]. Briefly, the suspension of (+)-cyanidan-3-ol-treated cells, 4 µl/ml of dye mixture, containing 100 µg/ml acridine orange (Sigma, USA) and 100 µg/ml ethidium bromide (Sigma, USA) in PBS were added, and washed once with PBS. After staining, cells were visualized immediately under a fluorescence microscope (Nikon Eclipse Ti, Japan).

### Estimation of percentage (%) of DNA fragmentation

The cells were incubated with 25 and 35 µg/ml of CD-3 for 48 h at 37 °C. Percent DNA fragmentation in HepG2 cells was assayed by the method of Kurita-Ochiai [16]. Briefly, DNA from the cells was released into the lysis buffer [0.2% Triton X-100, 10 mM Tris, and 1 mM EDTA (pH 8.0)] by rupturing the nucleus. The supernatant received after centrifugation at 14,000 *g* for 15 min, contained fragmented DNA while the intact DNA was in the pellet. The amount of DNA in both the supernatant and pellet was estimated by diphenylamine (DPA) assay [17]. The percentage of DNA fragmentation was calculated as the ratio of DNA in supernatant and DNA in pellet.

### Detection of DNA fragmentation by agarose gel electrophoresis

The cells were incubated with 25 and 35 µg/ml of CD-3 for 48 h at 37 °C. Briefly, DNA from the cells was released into the lysis buffer [0.2% Triton X-100, 10 mM Tris, and 1 mM EDTA (pH 8.0)] by rupturing the nucleus. The DNA in the supernatant was extracted using a 25:24:1 (v/v/v) equal volume of phenol: chloroform: isoamyl alcohol. The DNA was precipitated with ethanol, air-dried and dissolved in TE buffer [5 mM Tris-HCl (pH 8.0) and 20 mM edetic acid containing RNase A (0.1 mg/ml; Sigma)]. The samples were analyzed electrophoretically on 1% agarose gel containing 0.1 µg/ml ethidium bromide (EtBr, Sigma) [18].

### Apoptosis Assay by Annexin V-FITC and Propidium Iodide (PI) Staining

Annexin V-propidium iodide double staining assay was done to quantify apoptosis in HepG2 cells using FACscan flow cytometer (Becton-Dickinson, USA). After 48 h of CD-3 treatment, cells were harvested, washed with ice-cold PBS, and resuspended in 200 µl of binding buffer (10 mM HEPES/NaOH pH 7.4, 140 mM NaCl, 2.5 mM CaCl_2_) and incubated with 5 µl of Annexin V-FITC for 10 min at room temperature in the dark. Samples were washed with binding buffer, resuspended in PBS, counterstained with 5 µg/ml PI, and were analyzed by flow cytometry to identify apoptotic cells. Cells without treatment were used as a negative control. Cells showing up as Annexin V^-^/PI^+^ were recognized as necrotic, that showing up as Annexin V^+^/PI^+^ were taken as late apoptotic or secondary apoptotic, whilst Annexin V^+^/PI^-^ cells were recognized as early or primary apoptotic cells [19].

### RNA purification and RT-PCR analysis

The expression of apoptosis-related genes, such as p53, mdm2, p65, c-jun, c-fos, bcl-2, bax, cytochrome-c, caspase-3, -7, -8 and -9 were determined by RT-PCR. Total RNA isolation was done using TRI-REAGENT (Molecular Research Centre Inc. Ohio, USA). Single-strand cDNA was synthesized from 2 μg total RNA using moloney murine leukemia virus reverse transcriptase (M-MLV RT). RT-PCR was done using one step method of RT–PCR kit (QIAGEN, Germany). Briefly, 3 µg of RNA template from different groups was used in RT-PCR reaction. The 2.5 µl of 5X QIAGEN one step RT-PCR buffer was added followed by addition of 0.5 µl of dNTP mix (containing 10 mM of each dNTP). The 1 µl of each sense and antisense gene specific primers (from 10 µM stock) was added. Then 2µl QIAGEN one step RT-PCR enzyme mix and 1µl RNAase inhibitor (1U/µl) was added. Finally, PCR grade RNAase free water was added to make total volume 25 µl. Mixed it gently by vortex and centrifuged to collect all the components at the bottom of the PCR tubes. After an initial denaturation step of 1 min at 94 °C, 39 amplification cycles were performed. Each cycle included an initial denaturation step at 94 °C for 1 min, annealing for 45s and extension at 72 °C for 2 min. A final extension step of 5 min at 72 °C was performed in order to complete the PCR reaction. The amplified products were analyzed on 1.5% agarose gel electrophoresis and densitometric analysis of bands was done by ImageJ Software (NIH, USA). The oligonucleotide primer pairs for RT-PCR used in the present study are presented in table S1.

### Protein quantification by ELISA

ELISA was carried out in nuclear extract to quantify the level of p53, p65, c-jun, bax, bcl-2 and caspase-3. The assay was standardized by revising the different concentration of antigens and antibodies. Wells were coated with antigens diluted in 100 µl of 0.05 M carbonate buffer (pH 9.6) and kept overnight at 4 °C in a humid chamber. Flicked the plate to remove the unbound antigen solution and wells were blocked with 1% bovine serum albumin (BSA) in 0.1 M phosphate buffered saline (pH 7.2) for 1 h at 37 °C. Flicked and wells were washed thrice with 200 µl of PBS containing 0.05% (v/v) Tween-20. Wells were then incubated with anti-p53, anti-p65 and anti-caspase-3 (1: 250), anti-c-jun and anti-bax (1: 500) and anti-bcl-2 (1: 600) primary antibodies, respectively, diluted in PBS (containing 0.05% Tween-20 and 1% BSA) and incubated for 2 h at 37 °C. Plate was again washed and incubated with anti-rabbit secondary antibody (1: 1000) for 2 h at 37 °C. Wells were washed further three times as described above and color was developed by addition of 2,2´-azino-di-(3-ethyl)-benothiozolinsulphonic acid (ABTS) reagent and absorbance was measured at 405 nm by ELISA reader.

### Animals

Male Balb/C mice, 6-7 weeks old, 26-27 gm, were purchased from Sanjay Biological Museum, Amritsar, Punjab and kept in the Departmental animal house with controlled conditions of temperature (23 ± 5°C), humidity (60 ± 5%) and light (12 h light-dark cycles). They were fed a standard diet and water. All animals were acclimatized for 1 week before experimentation. Five animals were housed in each polypropylene cage with sterile paddy husk (procured locally) as bedding. The animal care and handling was done according to the guidelines set by the World Health Organization (WHO), Geneva, Switzerland, and the Indian National Science Academy (INSA), New Delhi, India. The protocol was approved by Institutional Animal Ethical Committee for animal handling (CPCSEA No. -13/RKN/BNCP-06).

### Experimental design and tumor induction

After one week of acclimatization, the mice were randomly divided into three groups (*n* = 10). Mice of group 1 served as control and these animals were orally administered with olive oil (100 µl/ mouse). Liver tumor was induced in group 2 and 3 with *N*-nitrosodiethylamine (NDEA) (200 mg/kg, body weight) dissolved in olive oil and given once by intraperitonial route. After two weeks of NDEA administration, it was promoted through subcutaneous injection of carbon tetrachloride (CCl_4_; 3 ml/kg) thrice a week for six weeks [20]. Group 3 animals were treated as group 2 mice along with oral administration of 200 µl of (+)-cyanidan-3-ol at the dose level 200 mg/kg body weight using a blunt tipped cannula, thrice a week throughout the experiment. (+)-Cyanidan-3-ol was administered orally 1 week before the initiation of NDEA treatment in group 3. At the end of 20 weeks, the body weight of each animal was taken before sacrifice. The mice were fasted overnight and killed by cervical decapitation. The greyish-white hyperplastic nodules were identified from the surrounding reddish brown liver tissue. Blood samples were collected from a common carotid and allowed to clot before centrifugation at 1000 *g* for 10 min at 4 °C to separate serum. The liver tissue was isolated and washed twice with ice-cold 0.1M phosphate buffer saline, pH 7.4, blotted, dried and weighed. The relative liver weight was calculated as the percentage ratio of liver weight to the body weight. A small portion of the tissue was fixed in 10% formalin for histological examination. The remaining tissue was stored at −20 °C for biochemical estimations, mRNA expression studies and protein level quantification.

### Biochemical estimations

The activities of serum aspartate (AST) and alanine transaminases (ALT) were analyzed by the method of Reitman and Frankel [21] while alkaline phosphatase (ALP) and γ-glutamyltransferase (γ-GT) were estimated by methods of Bergmeyer [22] and Szasz [23] respectively. The total sialic acid (TSA) [24] and lipid associated sialic acid (LASA) [25] levels in the serum and liver tissue were also measured. In the liver tissue, malondialdehyde (MDA) [26], nitrite [27] and antioxidants such as superoxide dismutase (SOD) [28], catalase (CAT) [29], glutathione peroxidase (GPx) [30], glutathione reductase (GR) [31], glutathione-S-transferase (GST) [32], total thiol (T-SH) [33], reduced glutathione (GSH) [34], levels were determined. Protein thiols (PrPr-SHs) content was calculated from the difference between the values of T-SH and GSH and expressed as µmoles of PrPr-SH/mg protein.

### Histopathological assessment

Liver sections were made immediately from the liver of different groups of animals, fixed in 10% formalin, dehydrated in gradual ethanol (50–100%), cleared in xylene, and embedded in paraffin. Sections (4–5 µm thick) were prepared and the pathological changes were observed microscopically after staining with hematoxylin and eosin (H & E). These slides were examined microscopically.

### Immunohistochemistry

Formalin-fixed and paraffin-embedded liver tissue sections with a thickness of 4-5 µm were de-paraffinized using xylene, rehydrated and treated with 3% H_2_O_2_ in PBS. Antigen retrieval was done using 0.01% sodium-citrate buffer (pH 6.0) followed by blocking in PBST containing 0.1% BSA and 10% FBS. Primary antibody incubation (p53, p65 and c-jun; 1:100) was carried out overnight at 4 °C. Slides were washed and incubated with HRP-labeled secondary antibody (1 h, RT, 1:200). Slides were washed (PBS containing 0.1% Tween 20), color was developed using DAB+H_2_O_2_, counterstained with haematoxylin and mounted in DPX (Sigma-Aldrich, USA). Images were captured using microscope (Nikon Eclipse Ti, Japan).

### Statistical analysis

Data are presented as mean ± standard error of mean (SEM). Data is subjected to one-way analysis of variance (ANOVA; 95% confidence interval), followed by Tukey’s post hoc test for the determination of level of significance (Software prism 6.0) and results were considered significant, if *P* < 0.05.

## Results

### Effect of CD-3 on human peripheral lymphocytes

The cytotoxic effect of CD-3 on human peripheral lymphocytes was characterized by MTT assay. When different concentrations of CD-3 (10-100 μg/ ml) were added to normal lymphocytes, the CD-3 did not show any toxic effect to lymphocytes after 48 h (Figure S2).

### *In vitro* proliferation studies on human hepatocarcinoma (HepG2) cells

**Figure 2:**
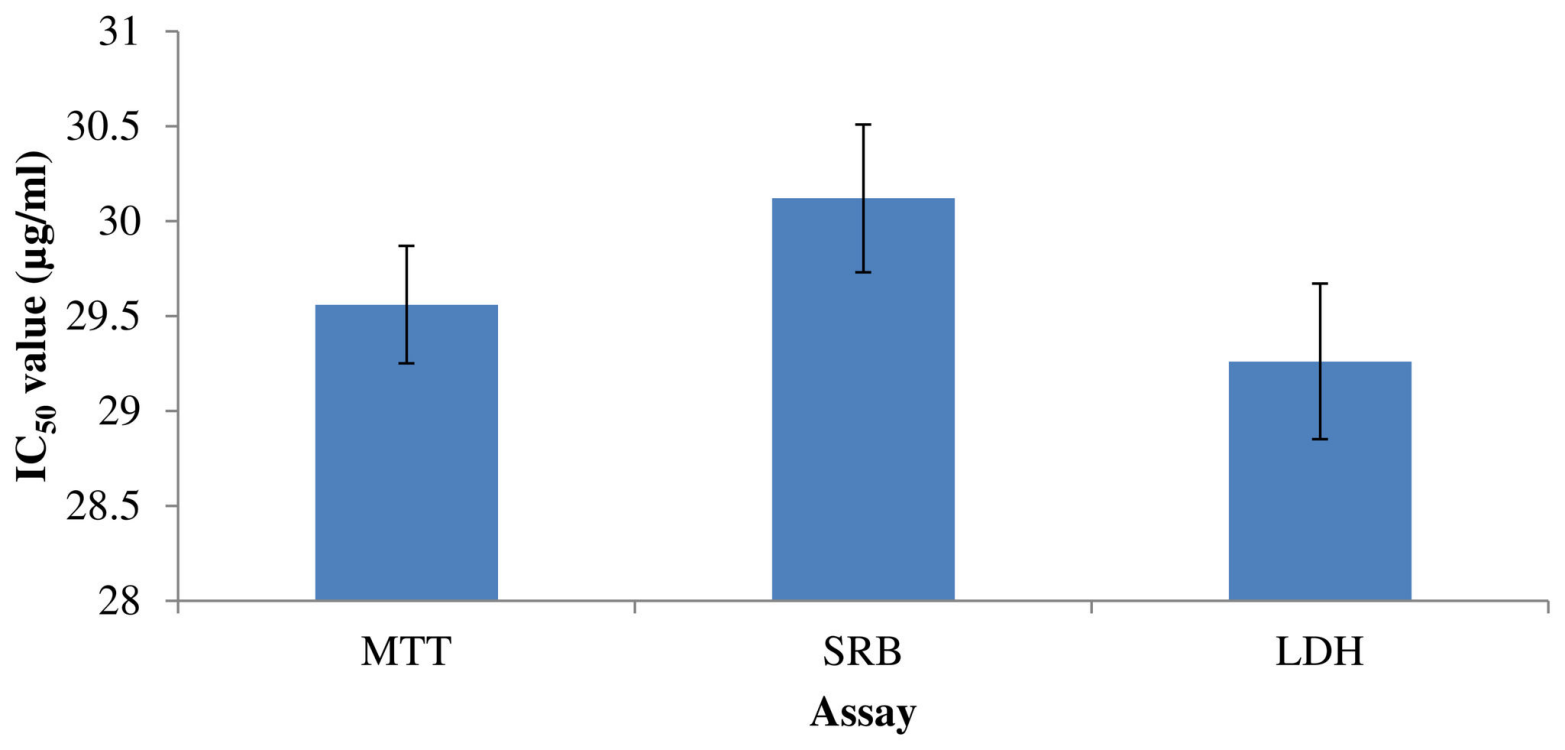
*In vitro* proliferation studies on human hepatocarcinoma (HepG2) cells. IC_50_value (μg/ml) of CD-3 was determined using MTT, SRB and LDH) leakage assays. Each value represents mean ± SEM of six replicates.

HepG2 cells were exposed to CD-3 for about 48 h and cytotoxicity was determined with different cytotoxic assays. The effect of CD-3 against HepG2 cells (concentration range 10–50 µg/ml) showed a decrease in percent cell viability in a dose-dependent manner, as compared with that of the control when examined by different cytotoxic assays. IC_50_ values (µg/ml) of CD-3 on HepG2 cells obtained by MTT, SRB and LDH assays are presented in Figure 2.

### Effect of (+)-cyanidan-3-ol on the morphology of HepG2 cells

**Figure 3:**
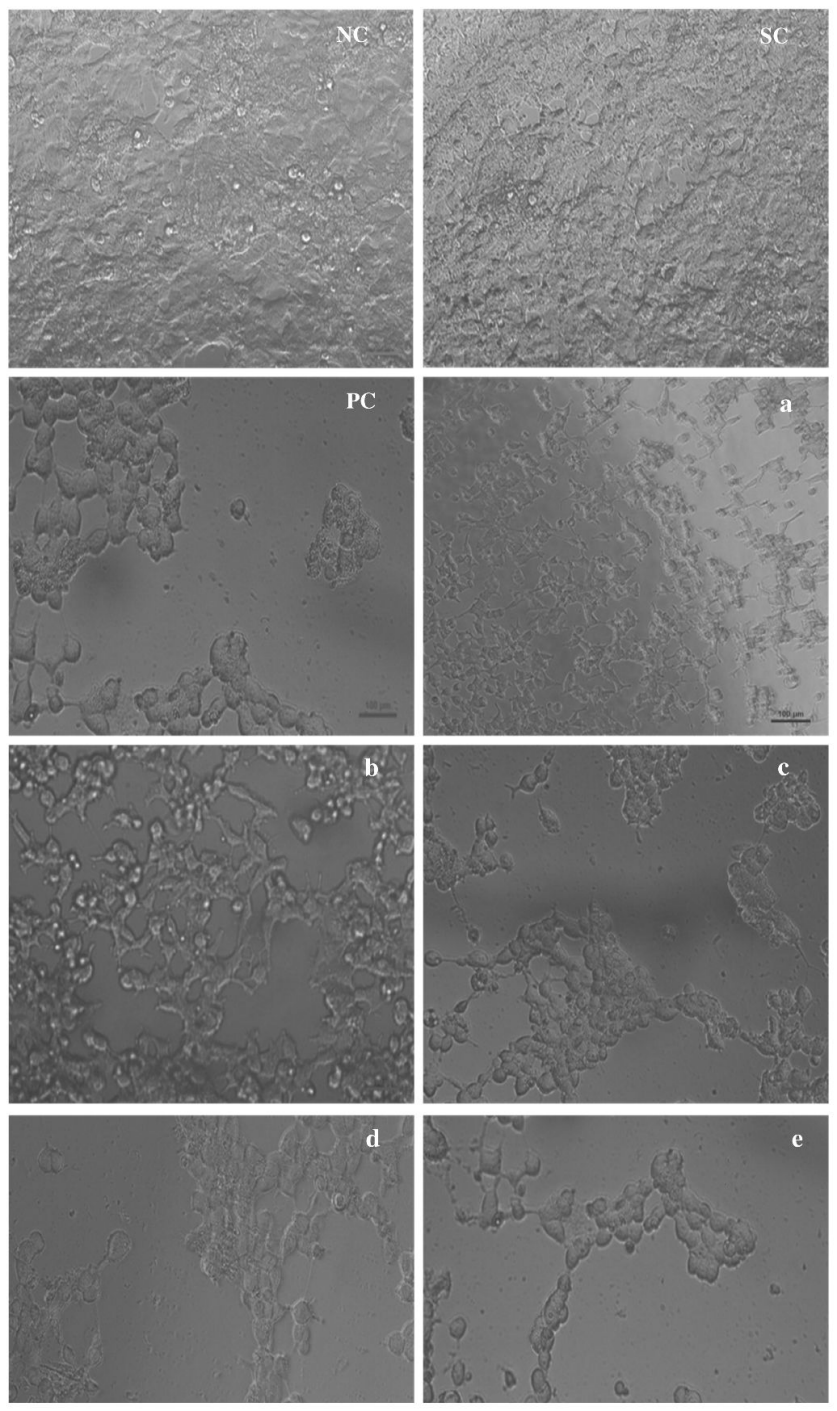
Morphological evaluation of HepG2 cells by phase-contrast inverted microscope. Cell line (HepG2) treated with CD-3 at a) 10 µg/ml, b) 20 µg/ml, c) 30 µg/ml, d) 40 µg/ml, and e) 50 µg/ml. *NC* negative control, *SC* solvent control, *PC* positive control-cisplatin 10 µg/ml.*Scale bars* 100 µm.

Direct observation of the morphological changes of HepG2 cells revealed growth inhibition and induction of cell death after treatment with CD-3. The morphological changes of HepG2 cells treated with 10, 20, 30, 40 and 50 µg/ml CD-3 for 48 h compared with the untreated cells. Formation of blebs on the cell membrane surface and protrusion of microspikes from the cells confirmed the occurrence of apoptosis. Cells undergoing apoptosis also displayed other types of morphological changes such as rounded up cells that shrink and lose contact with neighboring cells. The untreated cells showed a high confluency of monolayer cells (Figure 3) compared to CD-3-treated cells, which showed a reduction in cell volume and cell shrinkage in a dose dependent manner (Figure 3a–e).

**Figure 4:**
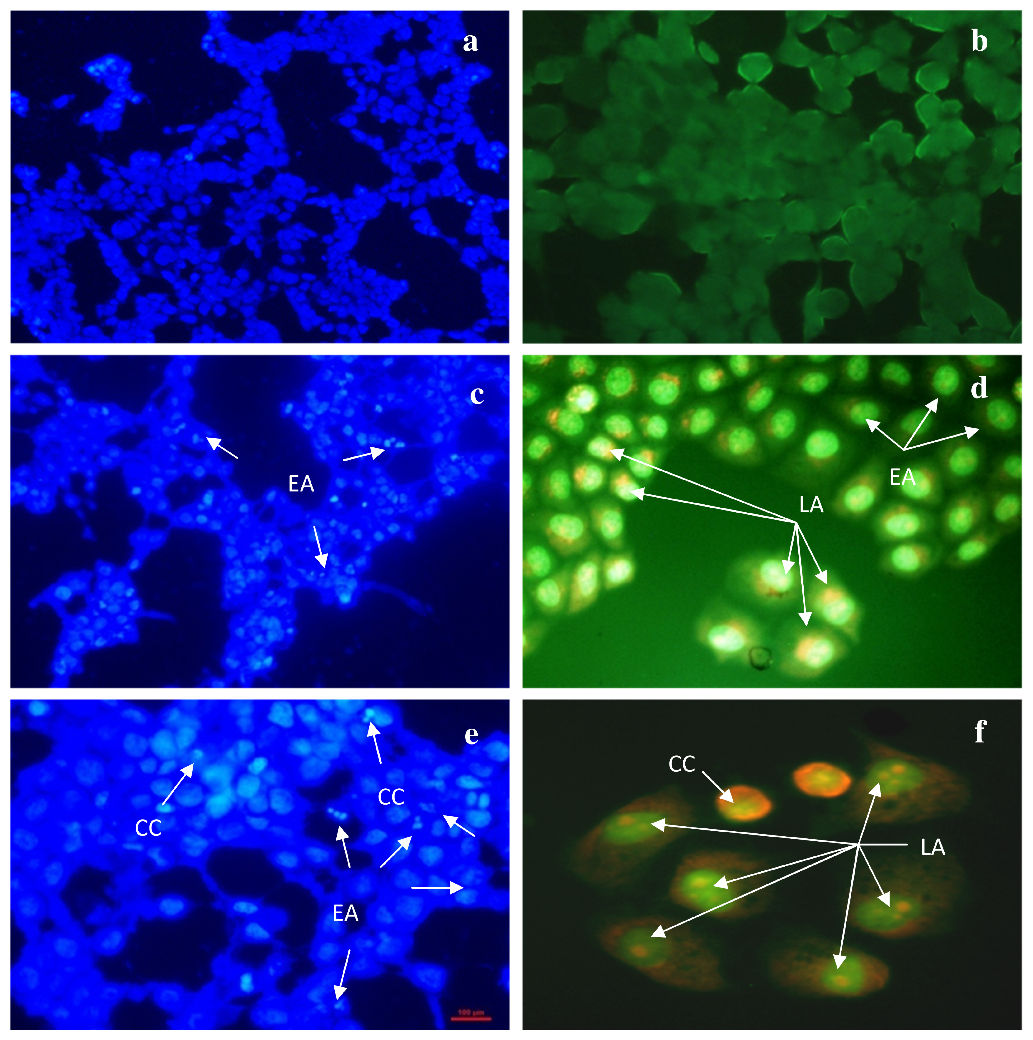
Apoptosis assay by Hoechst and acridine orange/ ethidium bromide. a and b) HepG2 control cells. c and d) HepG2 cells treated with 25 µg/ml of CD-3. e and f) HepG2 cells treated with 35 µg/ml of CD-3. Early apoptosis (EA) cells with apoptotic nuclei stained by Hoechst with fluorescence in blue spectrum (arrow); Chromatin condensation (CC); Late apoptosis (LA) stained acridine orange and ethidium bromide (AO/ EB) shown fluorescence in green and yellow/ orange spectrum (arrow).

To test whether the decrease in cell viability observed after treatment with CD-3 is due to apoptosis, HepG2 cells were stained with Hoechst 33258 (HO) and acridine orange/ethidium bromide (AO/EB) dye after exposure to 25 and 35 µg/ml CD-3. As shown in Figure 4, Hoechst staining, which correlated with the presence of cells with typical apoptotic nuclear morphology (nuclear shrinkage, DNA condensation and fragmentation), was present in the cells treated with different concentrations of CD-3 for 48 h (Figure 4c and e), but not in the non-treated controls (Figure 4a).

The morphological changes of the HepG2 cells-treated with CD-3 for 48 h at 25 and 35 µg/ml were also analyzed by acridine orange/ethidium bromide (AO/EB) fluorescence staining. AO/EB staining showed a concentration-dependent increase of apoptosis on treatment with CD-3, relative to the negative control. Cells stained green are viable cells, whereas, with fragmented green nucleus represented early apoptotic cells, and yellow/green dots of condensed nuclei were of late apoptosis. Cells treated with CD-3 exhibited characteristic changes of apoptosis e.g. cell shrinkage, nuclear condensation, fragmentation and formation of apoptotic bodies. Cells treated with 25 µg/ml of CD-3 showed signs of early apoptosis (Figure 4d). On the other hand, cells treated with 35 μg/ml of CD-3 showed similar features but with extra features of late apoptosis with formation of apoptotic bodies (Figure 4f).

### Effect of (+)-cyanidan-3-ol on DNA fragmentation in HepG2 cells

**Figure 5:**
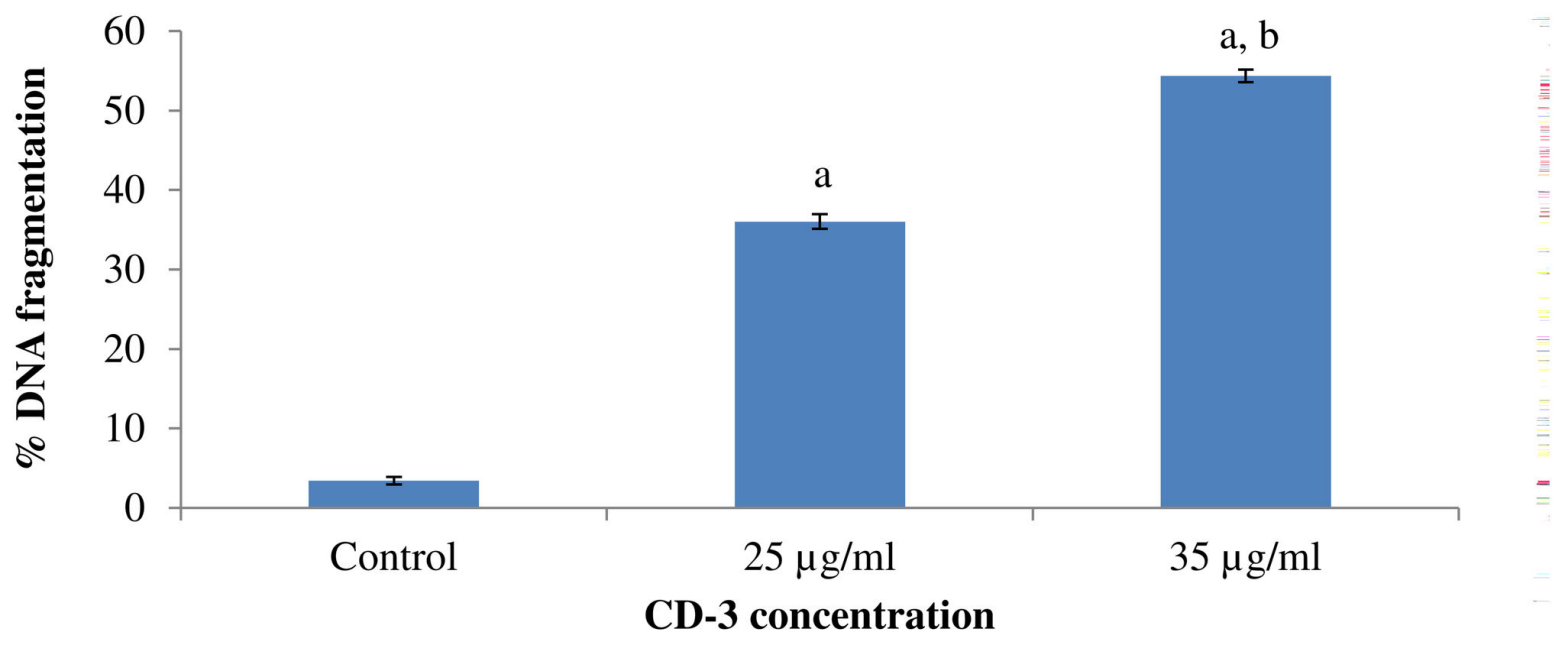
Effect of CD-3 on DNA fragmentation in HepG2 cells. Values are mean ± SEM of three observations. ^a^ *P* < 0.0001 w.r.t. control, ^b^ *P* < 0.0001 w.r.t. 25 µg/ml CD-3.

**Figure 6:**
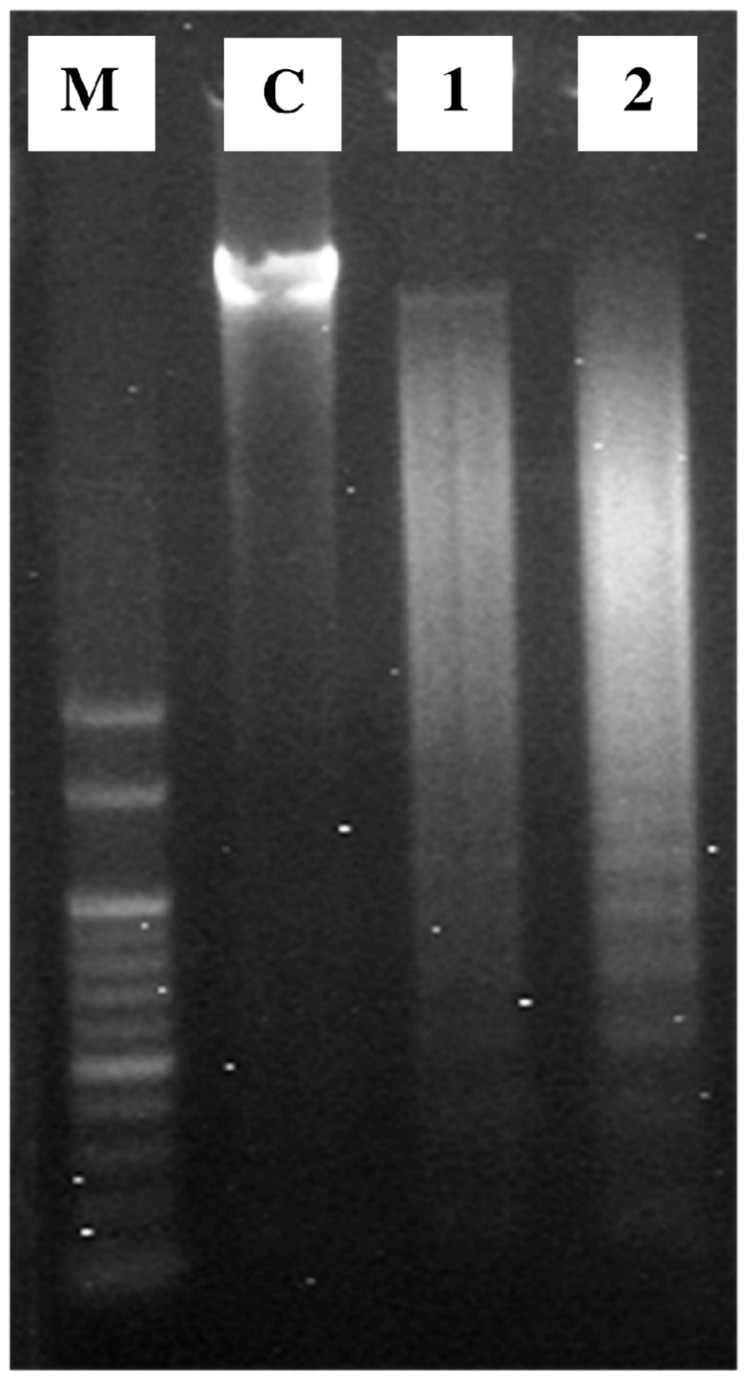
DNA fragmentation analysis by agarose gel electrophoresis. Lane M: DNA ladder (100 bp); Lane C: Control; Lanes 1, and 2: cells treated with 25 and 35 μg/ml CD-3, respectively.

An important feature of cell apoptosis is the fragmentation of genomic DNA. To elucidate whether CD-3 decreased cell survival by induction of DNA fragmentation, genomic DNA was isolated from HepG2 cells following exposure to 25 and 35 µg/ml of CD-3 and the percent DNA fragmentation was estimated. CD-3 treatment led to DNA fragmentation in HepG2 cells in a dose dependant manner in comparison, the DNA from untreated cells (Figure 5). In addition, these results were also confirmed by agarose gel electrophoresis of DNA. As shown by the characteristic DNA laddering in agarose gels, apoptosis was induced at CD-3 concentrations of 25 and 35 µg/ml (Figure 6).

### Flow cytometry analysis

**Figure 7:**
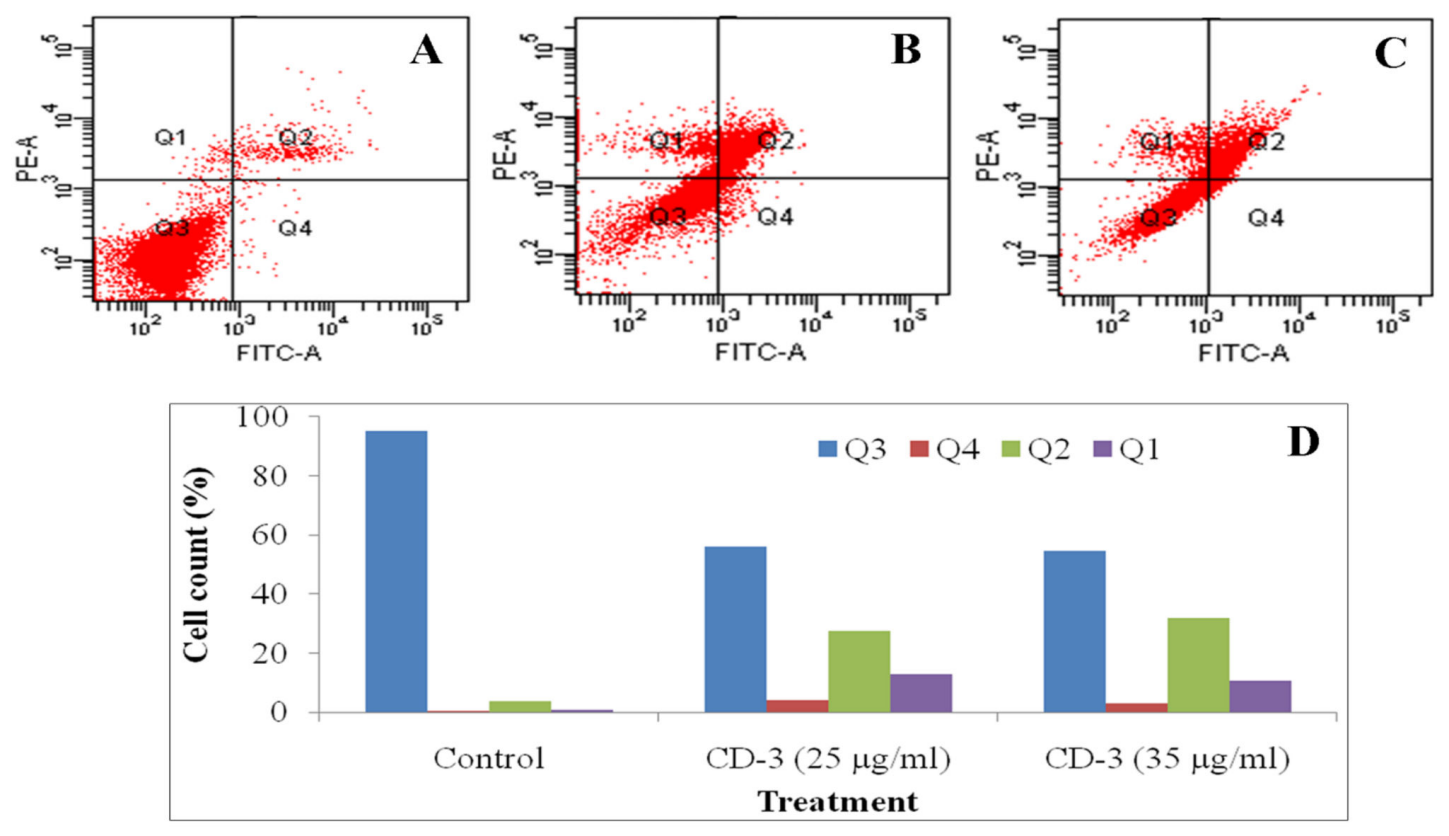
Apoptosis was assessed by flow cytometry after cells were stained with Annexin V-FITC and PI. (A) Control cells; (B–C) Cells treated with 25 and 35 µg/ml CD-3, respectively. (D) The summarized the percentage of cells in each quadrant. Q3: Living cells; Q4: Early apoptotic cells; Q2: Late apoptotic cells; Q1: Dead/Necrotic cells.

To further confirm that CD-3 induces cell apoptosis, we also used flow cytometry analysis with annexin V-FITC and PI double staining. HepG2 cells underwent apoptosis after exposure to CD-3 at 25 and 35 μg/ml for 48 h. The percentage of apoptotic cells stained by Annexin V-FITC is shown in Figure 7.

### mRNA expression studies in HepG2 cells by RT-PCR

**Figure 8:**
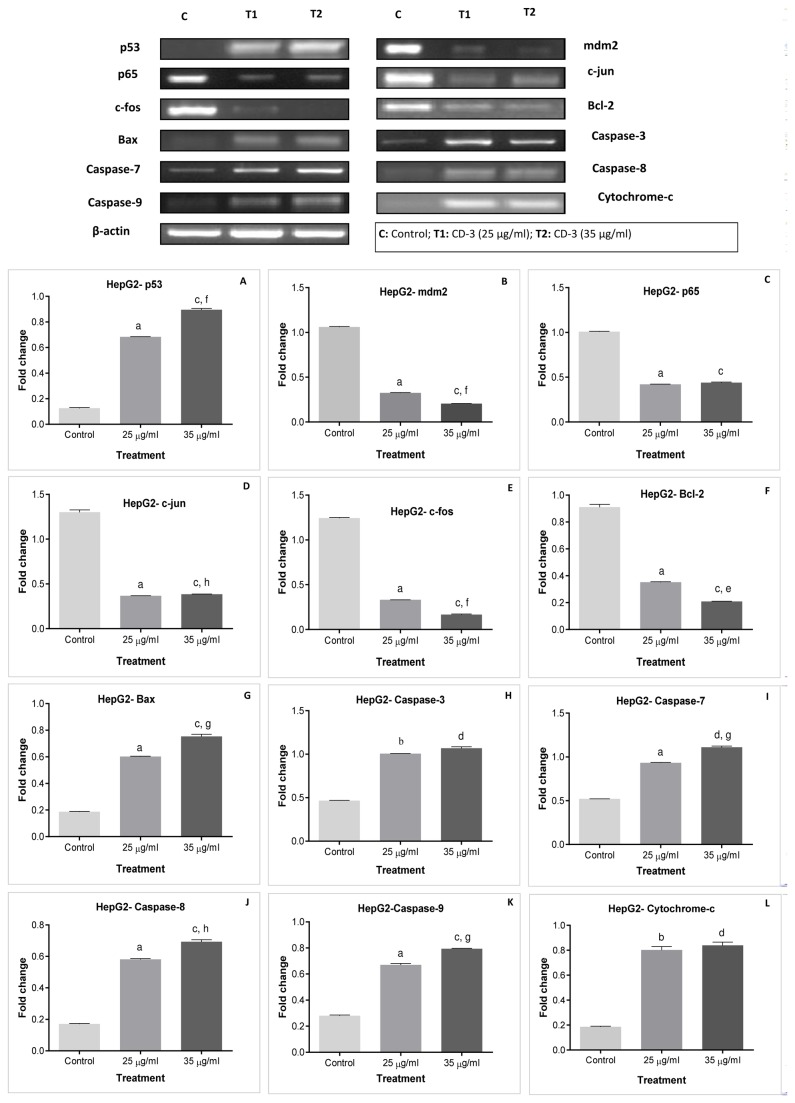
Expression of apoptosis-related genes in HepG2 cells after CD-3 treatment. mRNA expressions of β-actin, p53 (wild) (A), mdm2 (B), p65 (C), c-jun (D), c-fos (E), bcl-2 (F), bax (G), caspase-3 (H), -7 (I), -8 (J), and -9 (K), and cytochrome-c (L) and their densitometric analysis in HepG2 cells. Values are mean ± SEM of four independent observations. ^a^ *P* < 0.0001 w.r.t. control, ^b^ *P* < 0.001 w.r.t. control, ^c^ *P* < 0.0001 w.r.t. control, ^d^ *P* < 0.001 w.r.t. control, ^e^ *P* < 0.0001 w.r.t. 25 µg/ml, ^f^ *P* < 0.001 w.r.t. 25 µg/ml, ^g^ *P* < 0.01 w.r.t. 25 µg/ml, ^h^ *P* < 0.05 w.r.t. 25 µg/ml.

To elucidate cell signaling pathway activated by CD-3 in HepG2 cells, RT-PCR analyses was used to measure the mRNA expression of p53, mdm2, p65, c-jun, c-fos, bcl-2, bax, caspase-3, -7, -8 and -9 and cytochrome-c as well as the expression of the β-actin (an internal standard). The densitometric analysis of mRNA expression in each group was also carried and statistically compared (Figure 8).

A significant increase (*P* < 0.0001) in mRNA expression levels of p53, bax, caspase-7, -8 and -9 was observed in 25 and 35 µg/ml CD-3 treated HepG2 cells when compared to control HepG2 cells. Also the expression of p53 (*P* < 0.001), bax (*P* < 0.01), caspase-7(*P* < 0.01), -8 (*P* < 0.05) and -9 (*P* < 0.01) was significantly increased in 35 µg/ml CD-3 treated HepG2 cells in comparison to 25 µg/ml CD-3 treated HepG2 cells (Figure 8). A significant increase in the mRNA expression level caspase-3 was observed in 25 µg/ml CD-3 treated HepG2 cells in comparison to control HepG2 cells. Whereas, mRNA expression levels of caspase-3, was significantly increased (*P* < 0.0001) in 35 µg/ml CD-3 treated HepG2 cells in comparison to control HepG2 cells. However, no significant difference was observed in the expression of caspase-3 in 25 and 35 µg/ml CD-3 treated HepG2 cells (Figure 8). The expression of cytochrome-c was significantly increased (*P* < 0.001) in 25 and 35 µg/ml CD-3 treated HepG2 cells when compared to control HepG2 cells. No significant difference was observed in the expression of cytochrome-c in 25 and 35 µg/ml CD-3 treated HepG2 cells (Figure 8). A significant decrease (*P* < 0.0001) in mRNA expression levels of mdm2, p65, c-jun, c-fos and bcl-2 was observed in 25 and 35 µg/ml CD-3 treated HepG2 cells when compared to control HepG2 cells. Also the expression of mdm2 (*P* < 0.001), c-jun (*P* < 0.05), c-fos (*P* < 0.001) and bcl-2(*P* < 0.0001) was significantly decreased in 35 µg/ml CD-3 treated HepG2 cells in comparison to 25 µg/ml CD-3 treated HepG2 cells (Figure 8). No significant difference was observed in the expression of p65 in 25 and 35 µg/ml CD-3 treated HepG2 cells (Figure 8).

### Protein expression analysis of p53, p65, c-jun, bcl-2, bax and cleaved caspase-3 in HepG2 cells by ELISA

**Figure 9:**
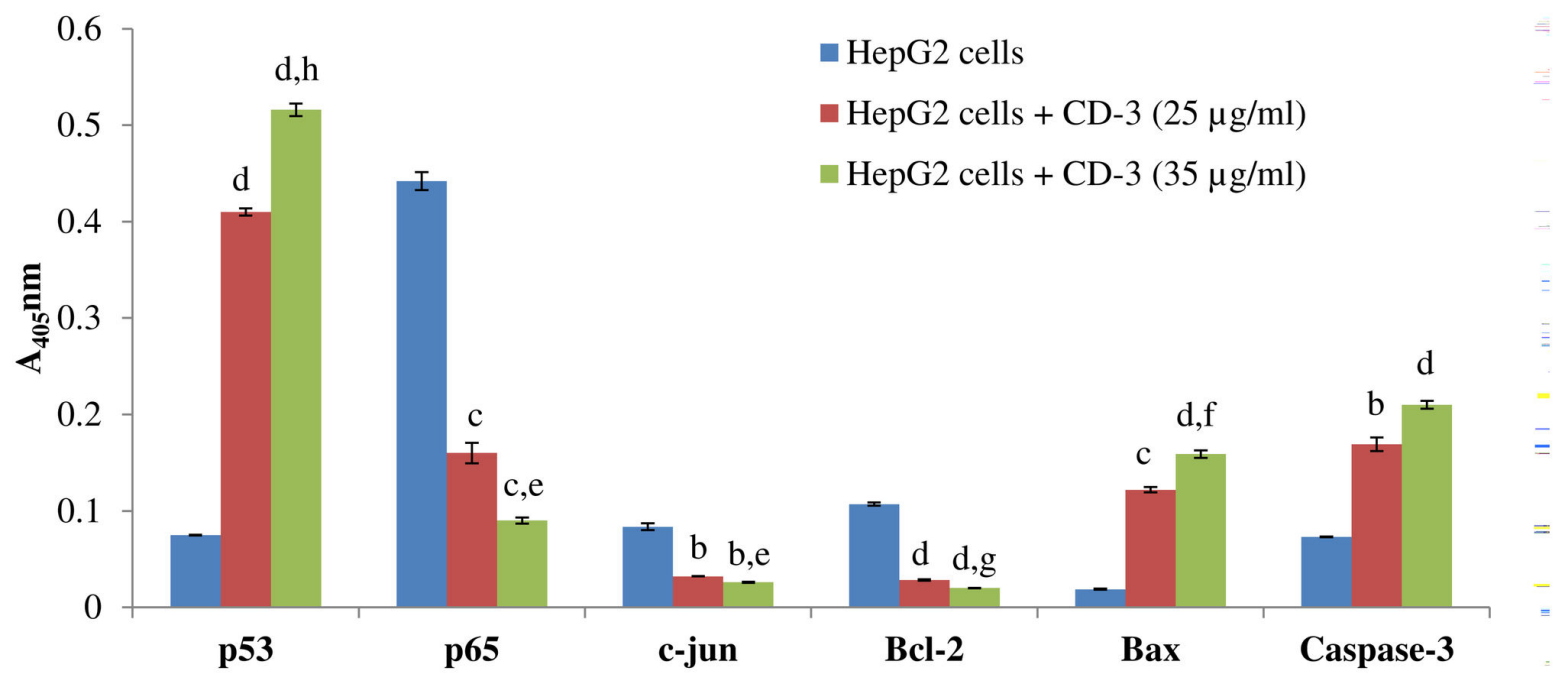
Quantitative protein expression in HepG2 cells by ELISA. Cells treated with 25 and 35 µg/ml of CD-3. Data represent the means ± SEM of six independent observations. ^a^ *P* < 0.05 w.r.t. control, ^b^ *P* < 0.01 w.r.t. control, ^c^ *P* < 0.001 w.r.t. control, ^d^ *P* < 0.0001 w.r.t. control, ^e^ *P* < 0.05 w.r.t. 25 µg/ml CD-3, ^f^ *P* < 0.01 w.r.t. 25 µg/ml CD-3, ^g^ *P* < 0.02 w.r.t. 25 µg/ml CD-3, ^h^ *P* < 0.001 w.r.t. 25 µg/ml CD-3.

A significant increase in the protein levels of p53 (*P* < 0.0001), bax (*P* < 0.001) and caspase-3(*P* < 0.01) was observed in cells treated with 25 µg/ml CD-3 in comparison to control cells. However, cells treated with 35 µg/ml CD-3 showed a significant increase (*P* < 0.0001) in protein levels of p53, bax and cleaved caspase-3 in comparison to control cells. Also the protein levels of p53 (*P* < 0.001), bax (*P* < 0.01) were significantly increased in 35 µg/ml CD-3 treated HepG2 cells in comparison to 25 µg/ml CD-3 treated HepG2 cells. No significant difference was observed in the protein level of cleaved caspase-3 in 25 and 35 µg/ml CD-3 treated HepG2 cells. The protein levels of p65 (*P* < 0.001), c-jun (*P* < 0.01) and bcl-2(*P* < 0.0001) were significantly decreased in 25 µg/ml and 35 µg/ml CD-3 treated HepG2 cells in comparison to control cells. Also the protein levels of p65 (*P* < 0.05), c-jun (*P* < 0.05) and bcl-2(*P* < 0.02) were significantly decreased in 35 µg/ml CD-3 treated HepG2 cells in comparison to 25 µg/ml CD-3 treated HepG2 cells (Figure 9).

### *In vivo* studies

The multiplicity and the incidence of the macroscopic lesions were calculated and the effect of CD-3 on NDEA/CCl_4_ induced liver incidence and multiplicity is shown in Table 1. Multiplicity was calculated by dividing the macroscopic lesions number by the number of animals with lesions. The NDEA treated group showed 100% tumor incidence whereas, CD-3 treated groups showed a marked reduction of tumor incidence of 77.78%.

**Table 1:** Effect of CD-3 on NDEA/CCl_4_-induced hepatocarcinogenesis.

**Group**	**Mice with lesions/ total of mice**	**Incidence (%)^a^**	**Multiplicity**	**Initial body weight (gm)**	**Final body weight (gm)**	**Liver weight (gm)**	**Relative liver weight (gm)**
Group 1	0/10	0.0	0.00	26.38 ± 0.19	27.51 ± 0.17	1.49 ± 0.009	5.43 ± 0.024
Group 2	6/6	100	4.83	26.19 ± 0.15	25.26 ± 0.14	1.90 ± 0.013	7.54 ± 0.078
Group 3	2/9	22.22	1.5^*^	26.17 ± 0.12	26.48 ± 0.13^*^	1.59 ± 0.049^*^	6.02 ± 0.18^*^

Group 1: Control; Group 2: NDEA/CCl_4_; Group 3: NDEA/CCl_4_/CD-3. Values are presented as mean ± SEM of all animals per group. ^a^ percent mice with tumor; ^*^ Significantly different from group 2 (*P* < 0.001).

### Effect of CD-3 on body weight and relative liver weight

The body and liver weights of control and experimental groups of mice were described in Table 1. A significant decrease (*P* < 0.001) in final body weight was observed in NDEA treated group (group 2) in comparison to vehicle treated control mice (group 1). In CD-3-treated group 3, the final body weights increased (*P* < 0.001) at the dose of 200 mg/kg. In NDEA treated mice (group 2), the relative liver weight was significantly increased (*P* < 0.001) when compared with vehicle treated control animals (group 1). However, administration of 200 mg/kg CD-3 significantly brought down (*P* < 0.001) the relative liver weight; as compared to NDEA treated mice (group 2) (Table 1).

### Effect of CD-3 on liver injury markers

The effect of CD-3 on liver injury markers is shown in Table 2. NDEA-treated (group 2) animals showed significant increased (*P* < 0.001) serum ALT, AST, ALP and γ-GT as compared to control (group 1) animals. In contrast, the CD-3-treated (group 3) animals at 200 mg/kg significantly decreased (*P* < 0.001) ALT, AST, ALP and γ-GT as compared to group 2 animals.

**Table 2:** Effect of CD-3 on the activities of marker enzymes in the serum.

**Groups**	**Parameters**			
	**ALT (U/L)**	**AST (U/L)**	**ALP (U/L)**	**γ-GT (U/L)**
Group 1	61.32 ± 1.80	81.65 ± 3.92	210.63 ± 3.90	35.66 ± 1.41
Group 2	213.86 ± 4.25^*^	204.97 ± 2.51^*^	493.50 ± 8.08^*^	195.12 ± 4.63^*^
Group 3	131.46 ± 7.93^*^	156.28 ± 2.51^*^	290.71 ± 6.08^*^	111.81 ± 3.81^*^

Group 1: Control; Group 2: NDEA/CCl_4_; Group 3: NDEA/CCl_4_/CD-3. Data represent the means ± SEM in each group. ^*^ *P* < 0.001 versus vehicle treated and NDEA/CCl_4_ or NDEA/CCl_4_/CD-3. Comparisons were made between Group 1 and Group 2 mice and between Group 2 and Group 3 mice.

### Effect of CD-3 on the TSA and LASA levels in serum and liver tissue

The results of TSA and LASA levels in the serum and liver tissue are depicted in Table 3. In serum, TSA and LASA levels were increased significantly (*P* < 0.001) in NDEA-treated animals (group 2) as compared to control animals (group 1). CD-3 treatment significantly reduced TSA (*P* < 0.001) and LASA (*P* < 0.001) levels as compared to animals treated with NDEA only. In liver tissue, the animals treated with NDEA only showed a significant increase (*P* < 0.001) in TSA and LASA levels as compared to control animals. On CD-3 treatment, the TSA and LASA levels were found to be decreased significantly (*P* < 0.001) as compared to NDEA-treated animals.

**Table 3:** Effect of CD-3 on TSA and LASA levels.

**Group**	**Parameters**			
	**TSA levels**		**LASA levels**	
	**Serum (µg/ml)**	**Liver tissue (µg/ml)**	**Serum (µg/ml)**	**Liver tissue (µg/ml)**
Group 1	1.91 ± 0.075	0.563 ± 0.038	0.599 ± 0.017	0.175 ± 0.003
Group 2	5.71 ± 0.19^*^	1.19 ± 0.042^*^	1.77 ± 0.065^*^	0.711 ± 0.018^*^
Group 3	2.67 ± 0.10^*^	0.793 ± 0.031^*^	0.966 ± 0.028^*^	0.303 ± 0.013^*^

Group 1: Control; Group 2: NDEA/CCl_**4**_; Group 3: NDEA/CCl_**4**_/CD-3. Data represent the means ± SEM in each group. ^*^ *P* < 0.001 versus vehicle treated and NDEA/CCl_**4**_ or NDEA/CCl_**4**_/CD-3. Comparisons were made between Group 1 and Group 2 mice and between Group 2 and Group 3 mice.

### Effect of CD-3 on the lipid peroxidation and nitrite levels in liver tissue

The results showed a significant increase in MDA levels in NDEA-treated animals (group 2) as compared to control animals (group 1) (*P* < 0.001; Table 4). On CD-3 treatment (group 3), a significantly decrease (*P* < 0.001) in MDA levels with respect to NDEA treated animals was observed. The nitrite levels were increased significantly in NDEA-treated animals (*P* < 0.001; Table 4). However, the animals treated with CD-3 showed a significant decrease (*P* < 0.001) in nitrite levels with respect to the animals treated with NDEA only.

**Table 4:** Effect of CD-3 on oxidative stress markers in mouse liver.

**Parameters**	**Group 1**	**Group 2**	**Group 3**
MDA (nmol/ mg protein)	3.38 ± 0.065	17.71 ± 0.32*	7.44 ± 0.31*
Nitrite (µg/mg protein)	0.500 ± 0.017	1.80 ± 0.031*	0.86 ± 0.025*
CAT (U/ mg protein)	5.26 ± 0.079	1.19 ± 0.038*	4.18 ± 0.053*
SOD (U/ mg protein)	18.55 ± 0.50	7.42 ± 0.85*	13.91 ± 0.55*
Total thiols	17.12 ± 0.20	6.39 ± 0.25*	13.59 ± 0.23*
GSH (nmol/ mg protein)	5.31 ± 0.16	1.69 ± 0.028*	3.98 ± 0.13*
PrPr-SH	11.81 ± 0.24	4.7 ± 0.23*	9.59 ± 0.23*
GST (GSH-CDNB conjugate/min/mg protein)	31.85 ± 0.70	9.59 ± 0.28*	24.26 ± 0.47*
GPx ((nmol NADPH oxidized/min/mg protein)	14.14 ± 0.40	3.95 ± 0.13*	9.28 ± 0.24*
GR ((nmol NADPH oxidized/min/mg protein)	15.15 ± 0.19	3.13 ± 0.13*	9.84 ± 0.26*

Group 1: Control; Group 2: NDEA/CCl_4_; Group 3: NDEA/CCl _4_/CD-3. Data represent the means ± SEM in each group. *P < 0.001 versus vehicle treated and NDEA/CCl_4_ or NDEA/CCl_4_/CD-3. Comparisons were made between Group 1 and Group 2 mice and between Group 2 and Group 3 mice.

### Effect of CD-3 on the antioxidants status in liver tissue

The results of the antioxidants levels are depicted in Table 4. A significant decrease in SOD and CAT activity were observed in NDEA-treated animals as compared to control animals (*P* < 0.001) in liver. On CD-3 treatment, a significant increase (*P* < 0.001) in SOD and CAT activities with respect to NDEA treated (group 2) animals was observed. A significant decrease (*P* < 0.001) in the levels of reduced GSH, T-SH, and PrPr-SH content in mice liver was observed in NDEA-treated (group 2) animals. Administration of CD-3 in group 3 animals caused a significant increase (*P* < 0.001) in GSH, T-SH and PrPr-SH levels (Table 4). On the other hand, levels of GST, GPx, and GR were significantly decreased in NDEA-treated group 2 animals (*P* < 0.001) (Table 4), whereas, in CD-3 treated group, a significant increase (*P* < 0.001) in the level of GST, GPx and GR was observed in comparison to NDEA-treated group.

### mRNA expression studies by RT-PCR in NDEA-induced hepatocarcinoma in Balb/c mice

**Figure 10:**
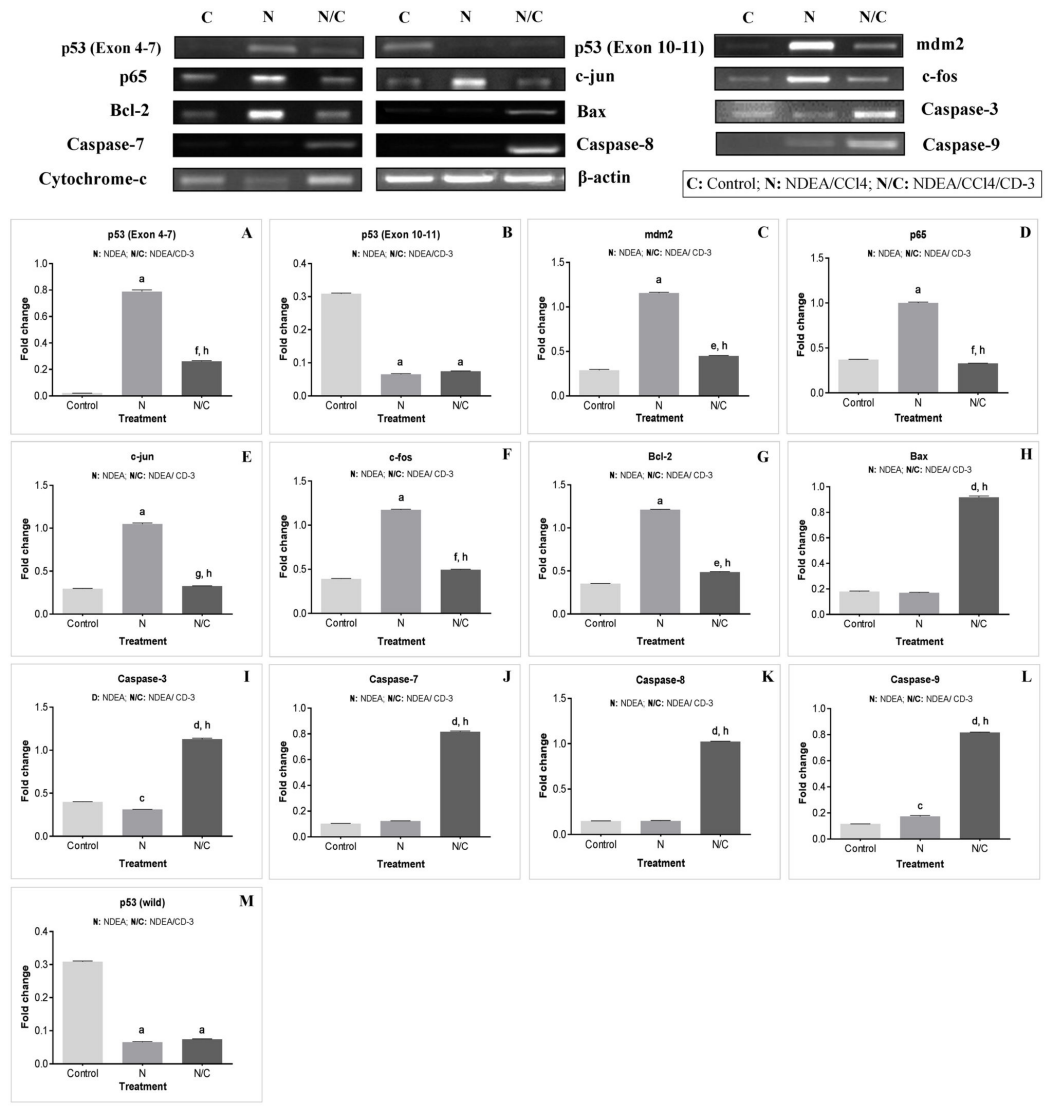
Expression of apoptosis-related genes in liver after CD-3 treatment. mRNA expressions of β-actin, p53 (mut) (A), p53 (wild) (B), mdm2 (C), p65 (D), c-jun (E), c-fos (F), bcl-2 (G), bax (H), caspase-3 (I), -7 (J), -8 (K), and -9 (L), and cytochrome-c (M) and their densitometric analysis in hepatocarcinoma. Values are mean ± SEM of four independent observations. ^a^ *P* < 0.0001 w.r.t. control, ^b^ *P* < 0.001 w.r.t. control, ^c^ *P* < 0.01 w.r.t. control, ^d^ *P* < 0.0001 w.r.t. control, ^e^ *P* < 0.001 w.r.t. control, ^f^ *P* < 0.01 w.r.t. control, ^g^ *P* < 0.05 w.r.t. control, ^h^ *P* < 0.0001 w.r.t. NDEA/CCl_4_ (group 2), ^i^ *P* < 0.001 w.r.t. NDEA/CCl_4_ (group 2).

To elucidate cell signaling pathway activated by CD-3 in NDEA-induced hepatocarcinoma in Balb/c mice, RT-PCR analyses was used to measure the mRNA expression of p53, mdm2, p65, c-jun, c-fos, bcl-2, bax, caspase-3, -7, -8 and -9 and cytochrome-c as well as the expression of the β-actin (an internal standard). In the present study, two different primers from different exonic regions of p53 gene were used for mRNA expression analysis. One primer was designed from exon 4-7 (Primer A) and the second primer was from exon 10-11 (Primer B). The densitometric analysis of mRNA expression in each group was also carried and statistically compared (Figure 10).

In case of primer A mRNA expression level the p53 was very low in vehicle treated control mice of group 1 animals which significantly increased (*P* < 0.0001) in NDEA-treated mice (group 2). However, CD-3 treatment in group 3 animals resulted in significant decreased (*P* < 0.0001) expression of p53 in comparison to NDEA treated liver tumors of group 2. On the other hand, mRNA expression level of p53 with primer B was high in vehicle treated control mice of group 1. However in NDEA-treated animals (group 2) the mRNA expression level of p53 was significantly decreased (*P* < 0.0001) in comparison to vehicle treated control mice of group 1. No significant difference was observed in mRNA expression level of p53 in CD-3 treated animals in comparison to NDEA treated liver tumors of group 2 (Figure 10).

A significant increase (*P* < 0.0001) in the expression of mdm2, p65, c-jun, c-fos and bcl-2 were observed in NDEA-treated mice (group 2) in comparison to vehicle treated control mice of group 1. CD-3 treatment in group 3 animals resulted in significant decreased (*P* < 0.0001) expression of mdm2, p65, c-jun, c-fos and bcl-2 in comparison to NDEA treated liver tumors of group 2. However, the expression of mdm2 (*P* < 0.001), p65 (*P* < 0.01), c-jun (*P* < 0.05), c-fos (*P* < 0.01) and bcl-2(*P* < 0.001) were significantly decreased in CD-3 treated group 3 mice in comparison to vehicle treated control mice of group 1 (Figure 10). A significant increase (*P* < 0.0001) in the expression of bax (*P* < 0.0001), caspase-3(*P* < 0.0001), -7 (*P* < 0.0001), -8 (*P* < 0.0001), -9 (*P* < 0.0001) and cytochrome-c (*P* < 0.001) were observed in CD-3 treated group 3 mice liver in comparison to NDEA-treated liver tumors (group 2). However, the expression of bax (*P* < 0.0001), caspase-3(*P* < 0.0001), -7 (*P* < 0.0001), -8 (*P* < 0.0001), -9 (*P* < 0.0001) and cytochrome-c (*P* < 0.0001) were significantly increased in CD-3 treated group 3 mice in comparison to vehicle treated control mice of group 1 (Figure 10).

### Protein expression analysis of p53, p65, c-jun, bcl-2, bax and cleaved caspase-3 in liver tissues by ELISA

**Figure 11:**
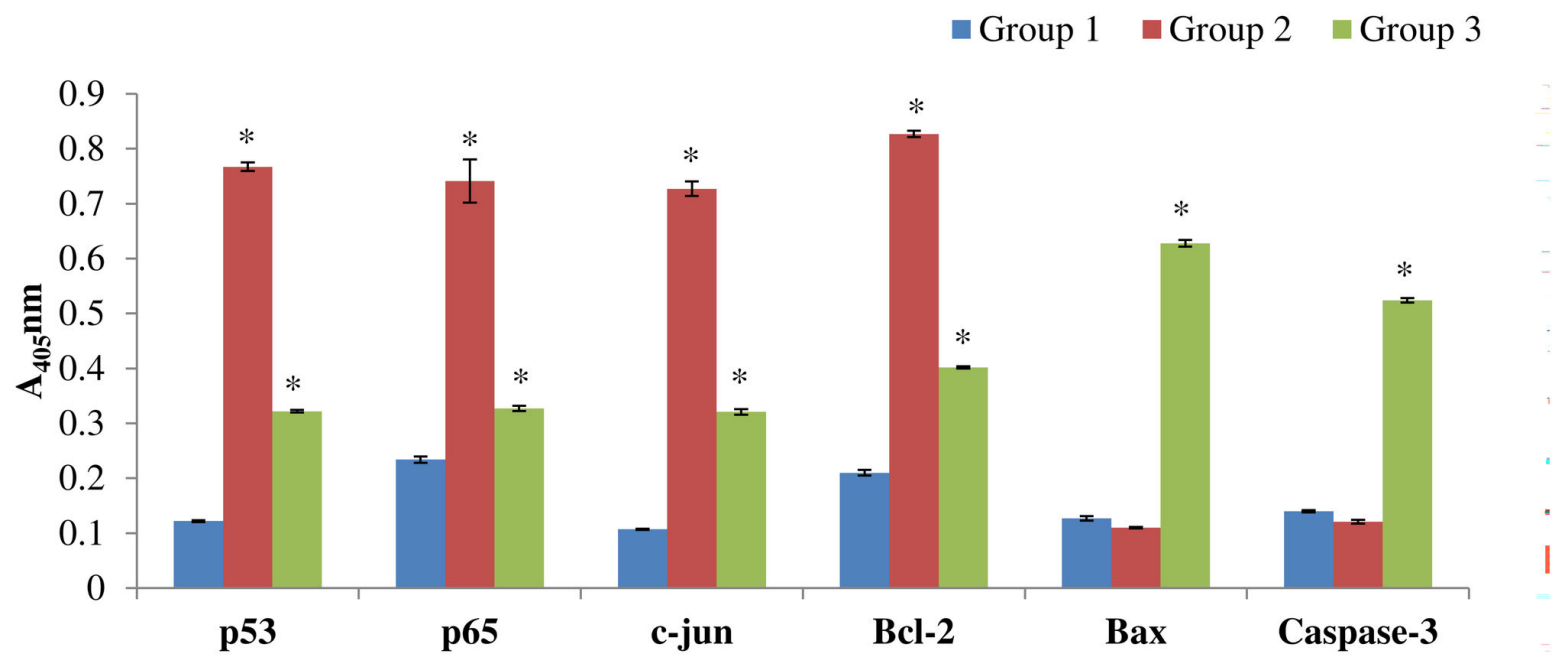
Quantitative protein expression in different groups by ELISA. Group 1: Control; Group 2: NDEA/CCl_4_; Group 3: NDEA/CCl_4_/CD-3. Data represent the means ± SEM of six independent observations. ^*^ *P* < 0.001 versus vehicle treated and NDEA/CCl_4_ or NDEA/CCl_4_/CD-3. Comparisons were made between Group 1 and Group 2 mice and between Group 2 and Group 3 mice.

There was a significant increase (*P* < 0.001) in the protein levels of p53, p65, c-jun and bcl-2were observed in NDEA-treated mice (group 2) as compared to vehicle treated control animals (group 1) whereas, a significant decrease (*P* < 0.001) was observed in the protein levels of p53, p65, c-jun and bcl-2 in CD-3 treated animals (group 3). The bax and cleaved caspase-3 protein levels were significantly increased (*P* < 0.001) in CD-3 treated mice (group 3) as compared to NDEA treated mice (group 2) (Figure 11).

### Histopathological observations

**Figure 12:**
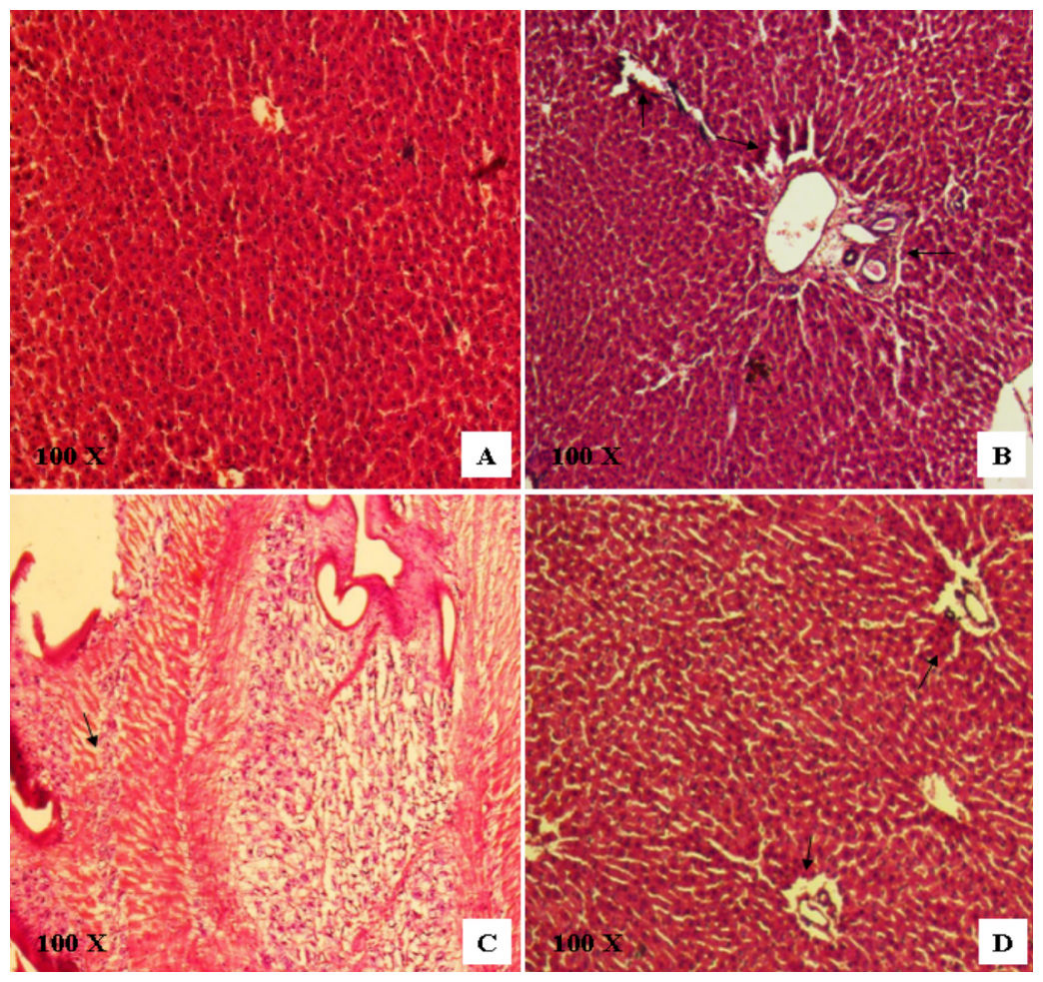
H & E stained sections of liver; 100 x. (A) Control group 1 revealed normal parenchymal cells with granulated cytoplasm and small uniform nuclei radically arranged around the central vein. (B & C) NDEA treated group 2 mice showing histology of a liver tumor that consists of hepatocytes only without portal tracts, congestion in the central vein and sinusoids, proliferation of Kupffer cells, and nodular hyperplasia of hepatocytes. The nuclei were mostly found to be pleomorphic with fine granular chromatin and occasionally with small nucleoli (B). The tumor architecture demonstrates severe centrilobular necrosis, degeneration in hepatocytes; extensive vacuolation was noticed in the cytoplasm encircling the nucleus. The sinuses were greatly dilated with hyperplastic kupffer cells (C). (D) NDEA/CD-3 treated group 3 mice showing normal cellular architecture with some hepatocytes showing few neoplastically transformed cells with minimal inflammatory cell infiltration around the portal triads.

The normal liver architecture with small uniform nuclei radically arranged around the central vein was observed in group 1 animal tissue (Figure 12A). Whereas, in group 2 (NDEA-treated mice) showed loss of cellular architecture. The tumor architecture demonstrated severe centrilobular necrosis, degeneration in hepatocytes, congestion in the central vein sinusoids, proliferation of Kupffer cells, and nodular hyperplasia of hepatocytes. Neoplastic cells were smaller than normal cells with granular cytoplasm and larger hyperchromatic nuclei (Figure 12B & C). Architecture of liver sections of CD-3 treated group 3 mice showed the features of a normal liver tissue with minimal inflammatory cell infiltration (Figure 12D).

### Immunohistochemistry

In order to study the alterations in the induction of various proteins, immunohistochemical localization of these proteins were carried out using immunohistochemistry (IHC). Negative control (without using specific antibody) from all the treatment groups did not show any detectable staining.

**Figure 13:**
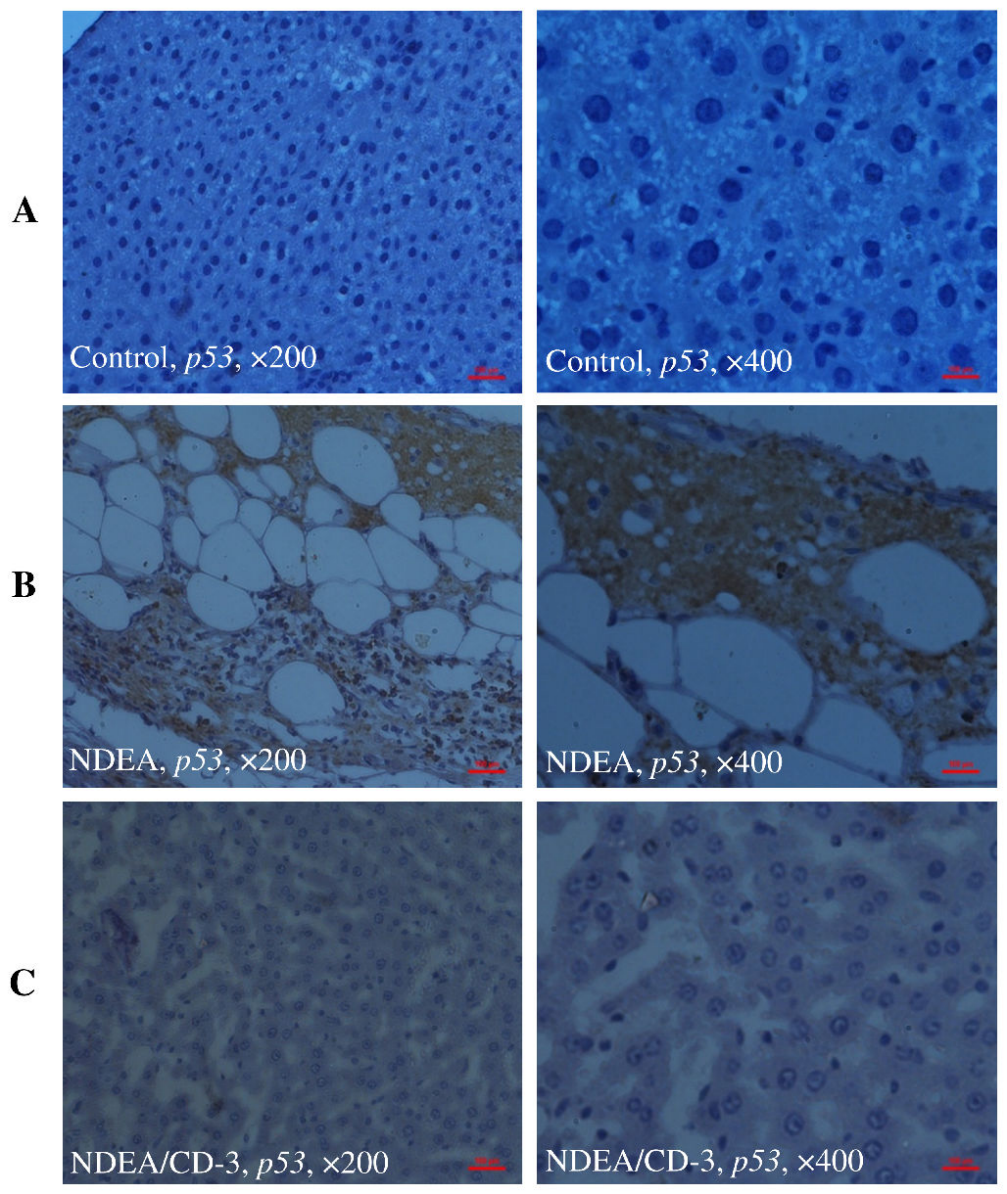
Immunohistochemical analysis of p53 expressions in mice liver. Panel ‘A’ photomicrographs shows liver section of control mice (group 1), Immunostained for p53 with specific anti-p53; Panel ‘B’ photomicrograph showing liver section of NDEA treated mice (group 2), Immunostained for p53 with specific anti-p53, showing intense staining throughout the lobules in hepatocytes, or in proliferating ductular cells; Panel ‘C’ photomicrograph showing liver section of NDEA/CCl_4_/CD-3-treated mice (group 3), Immunostained for p53 with specific anti-p53, showing less localization of p53 in hepatocytes. *Scale bar* 100µm.

**Figure 14:**
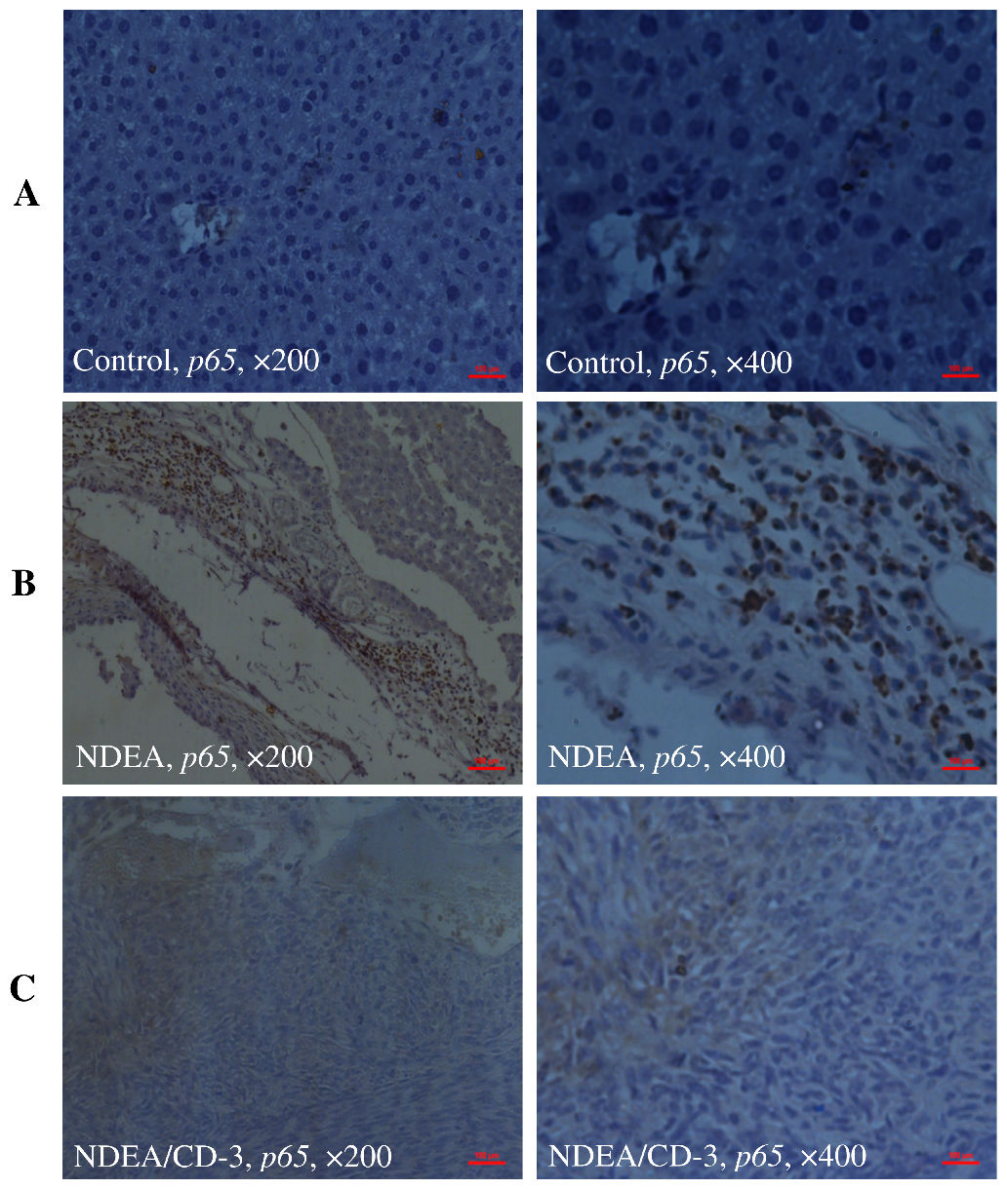
Immunohistochemical analysis of p65 expressions in mice liver. Panel ‘A’ photomicrographs shows liver section of control mice (group 1), Immunostained for p65 with specific anti-p65; Panel ‘B’ photomicrograph showing liver section of NDEA treated mice (group 2), Immunostained for p65 with specific anti-p65, showing intense staining of Kupffer cells; Panel ‘C’ photomicrograph showing liver section of NDEA/CCl_4_/CD-3-treated mice (group 3), Immunostained for p65 with specific anti-p65, showing less localization of p65 in hepatocytes. *Scale bar* 100µm.

**Figure 15:**
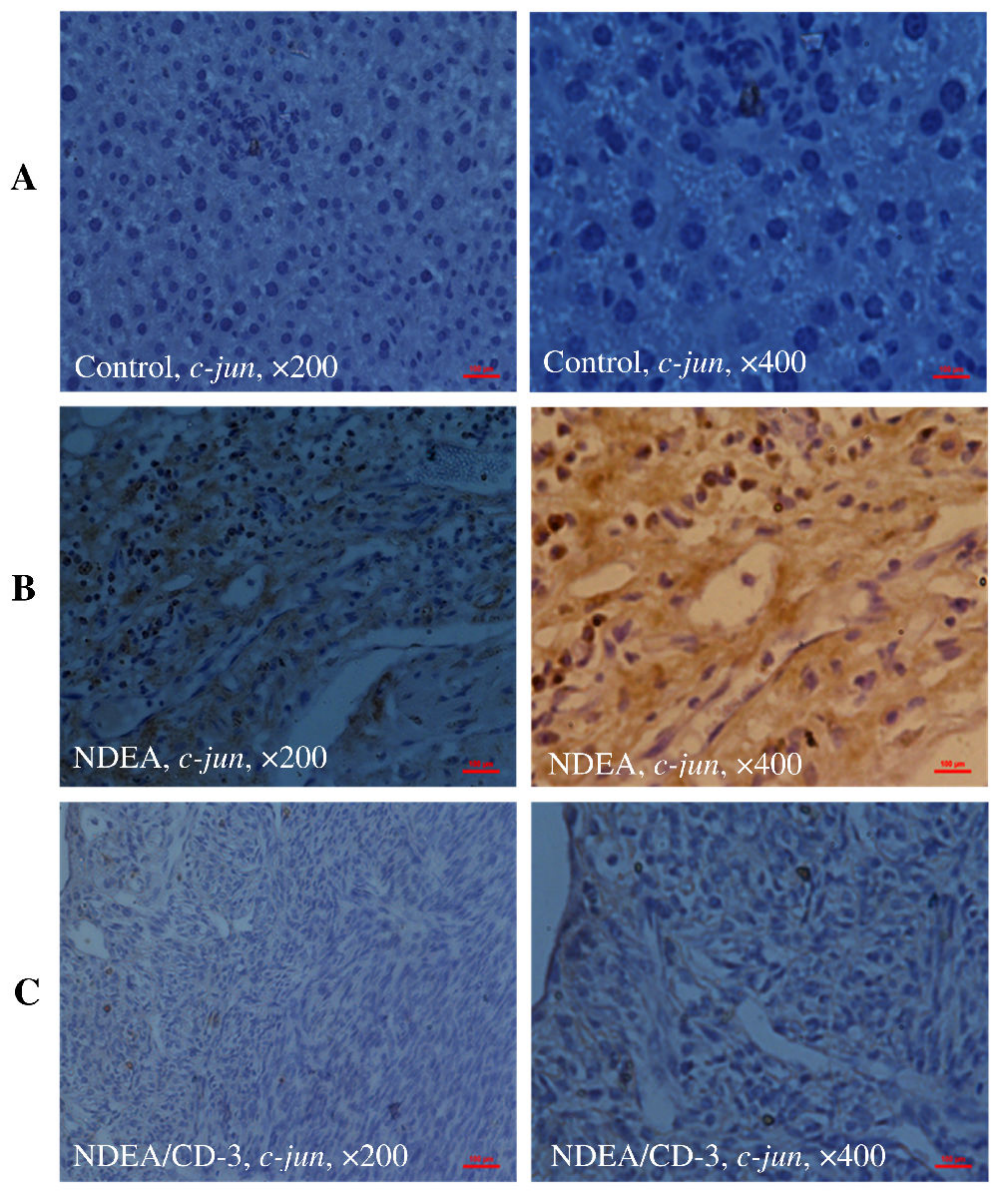
Immunohistochemical analysis of c-jun expressions in mice liver. Panel ‘A’ photomicrographs shows liver section of control mice (group 1), Immunostained for c-jun with specific anti-c-jun; Panel ‘B’ photomicrograph showing liver section of NDEA treated mice (group 2), Immunostained for c-jun with specific anti-c-jun, showing marked induction of c-jun throughout the lobules in hepatocytes, or in proliferating ductular cells; Panel ‘C’ photomicrograph showing liver section of NDEA/CCl_4_/CD-3-treated mice (group 3), Immunostained for c-jun with specific anti-c-jun, showing less localization of c-jun in hepatocytes. *Scale bar* 100µm.

The specific localization of p53, p65 and c-jun was studied in all the treatment groups. IHC investigation of control animals (group 1) revealed normal liver architecture with small uniform nuclei radically arranged around the central vein with no expression of p53 (Figure 13, panel A), p65 (Figure 14, panel A) and c-jun (Figure 15, panel A). In NDEA treated animals (group 2), intense localization of p53 (Figure 13, panel B), p65 (Figure 14, panel B) and c-jun (Figure 15, panel B) was seen throughout the lobules in hepatocytes, or in proliferating ductular cells as compared to respective control (group 1) which confirmed the increased expression of p53, p65 and c-jun in hepatocytes. In group 3 (NDEA/CD-3), a significant less localization of p53 (Figure 13, panel C), p65 (Figure 14, panel C) and c-jun (Figure 15, panel C) was seen in hepatocytes. The staining was although also visible in the hepatocytes but was faint as compared to NDEA treated group (group 2).

## Discussion

Apoptosis is a genetically programmed cell death mechanism that can be activated by various stimuli, including the activation of specific pro-apoptotic receptors, and cellular stress or injury caused by loss of growth factor signals, heat shock, irradiation, cytotoxic drugs, bacteria, and viruses. Disturbances in mechanisms that direct abnormal cells to undergo apoptosis perilously contribute to tumorigenesis; yield a logical target for potential therapeutic intervention [35].

This is the first report on the apoptosis induction and chemopreventive effect of CD-3 in HCC. Our data demonstrated that CD-3 could inhibit the proliferation of HepG2 cell line *in-vitro* via apoptosis induction in dose-dependent manner. The CD-3 also showed chemopreventive effect against NDEA/CCl_4_ induced HCC tumor growth *in-vivo*. We demonstrated that CD-3 dose-dependently inhibited the proliferation of HepG2 cells in various cytotoxicity assays. As a test to confirm the cytotoxicity of CD-3, the HepG2 cells were incubated with different concentrations of CD-3 and their morphological alterations were verified via a phase-contrast microscope. Results from the present study demonstrated that there were marked morphological changes in HepG2 cells following treatment with CD-3 which were indicative of cell apoptosis. The morphological changes that occurred in apoptotic cells were also perceived through fluorescence microscopy. It was possible to observe difference between early and late apoptosis in the HepG2 cells, showing that CD-3 exhibited a larger concentration response effect for late apoptosis and a smaller effect for early apoptosis, in both of the studied concentrations. Formation of DNA fragmentation is one of the characteristic features observed in apoptotic cells and it is generally considered as the biochemical hallmark of apoptosis [36]. The formation of a DNA ladder correlates with the early morphological signs of apoptosis and has been widely used as a distinctive marker of the apoptosis process [37]. The ladder-like appearance of DNA observed in the HepG2 cells treated with CD-3, was also another indicator of the apoptosis inducing capability of the CD-3. In addition, a noticeable phenomenon was that the percent DNA fragmentation was increased after treatment with CD-3 when analyzed by diphenylamine (DPA) assay. Data from an apoptosis assay showed that CD-3 induced obvious apoptosis in HepG2 cancer cells, presenting a concentration-dependent manner of apoptosis-specific fragmentation. This showed that CD-3 inhibited the proliferation of HepG2 cells via cell apoptosis. Flow cytometry analysis revealed that CD-3 treatment increased the apoptotic cells. These results suggested that the growth inhibition of HepG2 cells by CD-3 was due to its ability to induce apoptosis.

It is well documented that oxidative stress contributes to multiple physiological events including cell proliferation and inflammation, mediated by modifying redox sensitive AP-1 and NF-κB pathways. Oxidative stress occurs when the critical balance is disrupted due to excess production of ROS or depletion of antioxidants or both. During oxidative stress, malonaldehyde and other aldehydes are formed in the biological system as a result of lipid peroxidation. The products of LPO are considered mutagenic and carcinogenic as they cause damage to cellular macromolecules by generating ROS [38,39].

The current study showed an elevated level of MDA in liver of the animals treated with NDEA/CCl_4_ as compared to control animals. The increased level of lipid peroxidation may be due to the poor antioxidant defense or inactivation of antioxidant enzymes in cancerous tissues. A significant decrease in MDA levels by CD-3 treatment indicated their role in reducing oxidative stress, thus indicating its protective potential against HCC. Nitric oxide radical (NO·) plays an important role in physiological and pathological processes. An increased nitrite level is generally associated with the process of carcinogenesis [40]. The present results showed that nitrite levels, an indicator of NO·, were significantly decreased on CD-3 treatment. 

Body’s antioxidant defense systems operate for scavenging ROS to prevent the oxidative stress and antioxidant enzymes, namely, SOD and CAT acts as the first line of cellular defense against oxidative damage [41]. NDEA/CCl_4_ administration decreased the activities of these antioxidant enzymes in the liver tissue, which may be related to saturation of SOD with superoxide radicals in tumor cells or a decrease or loss of expression of SOD. A decrease in SOD activity will result in decreased production of H_2_O_2_ which in turn affects CAT activity. The second line of cellular defense includes glutathione antioxidant system that plays an important role against free radicals [42]. Reduced glutathione and its dependent enzymes, GPx, GR and GST serve as reliable markers of chemoprevention [43]. The present data showed significant decrease in the activities of GPx, GR, and GST in mice liver after NDEA/CCl_4_ exposure, resulting in considerable decline in the activities of these enzymes. In response to oxidative stress, GPx works in tandem with CAT to scavenge excess of H_2_O_2_ and lipid peroxides [44,45]. However, unlike CAT activity, GPx activity also depends on the balance between the levels of GSH and GSSG [46]. Thus, decrease in GPx activity may be implicated in both free radical dependant inactivation of enzyme [47] and depletion of its co-substrates, i.e., GSH and NADPH [48]. The observed decrease in GPx activity may also be due to reduced availability of GSH. A significant decrease in GR activity may be attributed to the impaired conversion of GSSG into GSH and thus balancing GSH/GSSG ratio [49]. Thiol groups have ability to act as reducing agents and thus play a central role in coordinating the antioxidant defense system [50]. The present findings revealed alterations in the levels of T-SH, GSH, and PrPr-SH in NDEA/CCl_4_-treated animals. The decrease in T-SH content might be contributed to reduction in GSH levels and/or change in PrPr-SH groups. The observed decrease in GSH levels may be due to diminished activity of GR, which is a crucial enzyme for maintaining GSH/GSSG ratio in the cell. A significant restoration in the activities of these enzymes after CD-3 administration indicated that CD-3 acts as an effective antioxidant.

Experimental evidence suggests that apoptosis can be mediated by number of different ways and that there are numerous regulatory molecules associated with those paths. p53 plays a key role in the process of apoptosis, DNA repair and tumor suppression pathways. Both extrinsic and intrinsic apoptotic pathways are activated by p53 [51]. Many chemopreventive agents are known to exert their anticancer effects through the induction of apoptosis via p53 dependent mechanisms. The inactivation of p53 causes genetic instability leading to the accumulation of genetic alterations, which induce malignant transformation of cells. In addition, it was found that mutant p53 protein that frequently accumulates in human tumors facilitates tumor initiation and progression by a “gain of function” mechanism [52,53]. In the present study, we observed the mRNA and protein expression levels of p53 in CD-3 treated HepG2 cells using RT-PCR and ELISA, respectively. We found that CD-3 markedly increased the mRNA and protein expression levels of p53 in HepG2 cells in a dose-dependent manner. However, in *in-vivo* study the analysis of our RT-PCR bands by using both p53 primer pairs, it appears that in mouse different isoforms were produced by splicing mechanism and their level of expression compare to each other decide the fate of the cell. We also suspect that in our case these different spliced products in cancerous tissue contain some alteration in 3ʹ-UTR region which somehow was related to apoptosis or inhibition of mdm2-p53 interaction. Moreover, in cancerous tissue exon 10 and 11 is known to contain two transcript variants so it is quite possible (but not certain) that second primer was not able to amplify some of the unknown spliced variant that may be produced at that area. Since, primer B position is responsible for transcript variant one and two and that might be the possible region behind low expression level of mRNA in cancerous tissue. Similarly, immunohistochemistry studies indicated intense localization of p53 throughout the lobules in hepatocytes, or in proliferating ductular cells in NDEA/CCl_4_-treated animals. However, less immunohistochemical localization of p53 was observed in CD-3 treated animals. Mutant p53 proteins accumulate to high levels in many cancer cells and the p53 protein and the p53 response to DNA damage represent key points for therapeutic intervention. The mdm2 gene encodes a negative regulator of the p53 tumor suppressor. Mdm2 may impart some of its tumorigenic properties by increasing the degradation of multiple cellular proteins [54]. In the present investigation, a significant increase in the mdm2 mRNA expression levels was seen in both HepG2 cells and NDEA/CCl_4_-treated animals. However, a significant decrease in mdm2 expression levels was observed on CD-3 treatment in both *in-vitro* and *in vivo* models. Mdm2 is the primary cellular inhibitor of p53 in cancers and targeting the Mdm2-p53 interaction is an attractive cancer therapeutic strategy [55].

One of the major gene groups that regulate apoptosis is the bcl-2 family such as bax and bcl-2 proteins which elicit opposing effects on mitochondria. Enhancement of pro-apoptotic Bax over Bcl-2 proteins can enhance the permeability of the mitochondrial membrane, which in turn results in the release of apoptogenic factors. Bcl-2, on the other hand, prevents this process by preserving mitochondrial integrity and blocks the release of soluble inter-membrane factors such as cytochrome c that activate the effectors of apoptosis [56]. Thus, it has been suggested that the ratio between the levels of pro-apoptotic and the anti-apoptotic factor determines whether a cell responds to an apoptotic signal. The current study indicated that, CD-3 up-regulated the mRNA and protein expressions of bax and down-regulated the mRNA and protein expression levels of bcl-2 in both HepG2 cells and NDEA/CCl_4_-treated animals. Also, mRNA expression level of cytochrome-c was increased in CD-3 treated HepG2 cells and NDEA/CCl_4_-treated animals. Hence, the ratio of pro-apoptotic proteins to the anti-apoptotic proteins was altered in favor of apoptosis. Thus, the results suggested that an up-regulation of bax and the corresponding down-regulation of bcl-2 proteins observed in this study may be one of the critical mechanisms through which CD-3 induced apoptosis.

The induction of apoptosis is almost always associated with the activation of caspases; a conserved family of enzymes that irreversibly commit a cell to die. The release of cytochrome c from mitochondria to cytosol after being induced by a variety of apoptosis-inducing agents leads to the formation of apoptosome which forms a platform for the efficient processing and activation of caspase-9. Activation of caspase-9, in turn, cleaves effectors caspases such as caspase-3 and 7 which eventually lead to apoptosis [57]. In the next series of experiment, we assessed the effect of CD-3 on the cascade of caspases. To investigate the effect of CD-3 on the caspase cascade, mRNA expression levels of caspase-3, -7, -8, and -9 and protein level of cleaved caspase-3 were determined in our experiment. Results from the present study demonstrated that mRNA expression levels of caspase-3, -7, -8 and -9 and protein level of cleaved caspase-3 were increased in a both HepG2 cells and NDEA/CCl_4_-treated animals on CD-3 treatment. The activation of caspases-3, -7 and -9 is a result of the induction of the intrinsic pathway, while activation caspase-8 and then caspase-3 and -7 may be the result of the induction of the extrinsic pathway. In both pathways, the initiator caspase cleaves and activates downstream effector caspases, such as caspase-3 and -7.

Activated protein-1 (AP-1) and nuclear factor-κB (NF-κB), two of important transcription factors, play important roles in signal transduction pathways of cell differentiation, proliferation and apoptosis in response to a variety of physiological and pathological stimuli [58,59]. AP-1 activation requires Jun and Fos through the formation of homo and hetero-dimers, and regulates transcription of a broad range of genes involved in cell differentiation and proliferation [60]. In the present study, we observed the mRNA expression levels of c-jun and c-fos and protein expression level of c-jun were markedly decreased on CD-3 treatment in both HepG2 cells and NDEA/CCl_4_-treated animals. These results were also supported by immonohistochemistry studies which had shown the marked induction of c-jun throughout the lobules in hepatocytes, or in proliferating ductular cells in NDEA/CCl_4_-treated animals. However, less immunohistochemical localization of c-jun was observed in animals treated with CD-3. NF-κB is thought to up-regulate expression of genes that cause suppression of the apoptotic response in cancer cells [59]. In the present study, a significant increase in the mRNA and protein expression levels of NF-κB (p65) was observed in both HepG2 cells and NDEA/CCl_4_-treated animals. However, a significant decrease in the mRNA and protein expression levels of p65 was observed on CD-3 treatment in both HepG2 cells and NDEA/CCl_4_-treated animals. Similarly, immunohistochemistry studies indicated intense localization of p65 in NDEA/CCl_4_-treated animals. However, weak immunohistochemical localization of p65 was observed in animals treated with CD-3. Several chemopreventive agents are inhibitors of NF-ĸB activation. These inhibitors can block any one or more steps in the NF-ĸB signaling cascade, the translocation of NF-ĸB into the nucleus, DNA binding of the dimers and interactions with the basal transcription activation. Thus, blockers of NF-κB should be beneficial not only in prevention but also in the treatment of cancer.

In conclusion, the present study demonstrated that CD-3 could inhibit the proliferation of HCC cell line *in-vitro* and suppresses HCC tumor growth *in-vivo* and consequently induced caspase-dependent apoptosis via up-regulation of p53, bax and down-regulation of bcl-2, AP-1 and NF-κB. Therefore, the results from this study provided critically important experimental facts to suggest that CD-3 may be a potential therapeutic agent for treating HCC.

## Supporting Information

Figure S1 The representative chromatogram showing the purity of CD-3 by analytical reverse phase HPLC. HPLC experimental conditions were: Instrument: Waters 515 with auto-sampler and the photo diode array (PDA) detector (Waters 2996); HPLC column: Waters spherisorb symmetry (C18 (5.0 μm) 250 mm × 4.6 mm); Mobile phases: 0.1% trifluoroacetic acid (TFA): acetonitrile (85: 15, v/v); Flow rate: 1.0 ml/min; Cycle time of analysis was 25 min at 30°C.
(TIF)

Figure S2 Effect of CD-3 on the percentage viability of normal lymphocytes. (TIF)

Table S1 The oligonucleotide primer pairs used in reverse transcription polymerase chain reaction. (DOCX)
