# Supplementary figures and images for: Growth Inhibition and Apoptosis Induction by (+)-Cyanidan-3-ol in Hepatocellular Carcinoma
Source: PLoS One. 2013 Jul 24;8(7):e68710. doi: 10.1371/journal.pone.0068710 (PMC3722203; doi:10.1371/journal.pone.0068710)

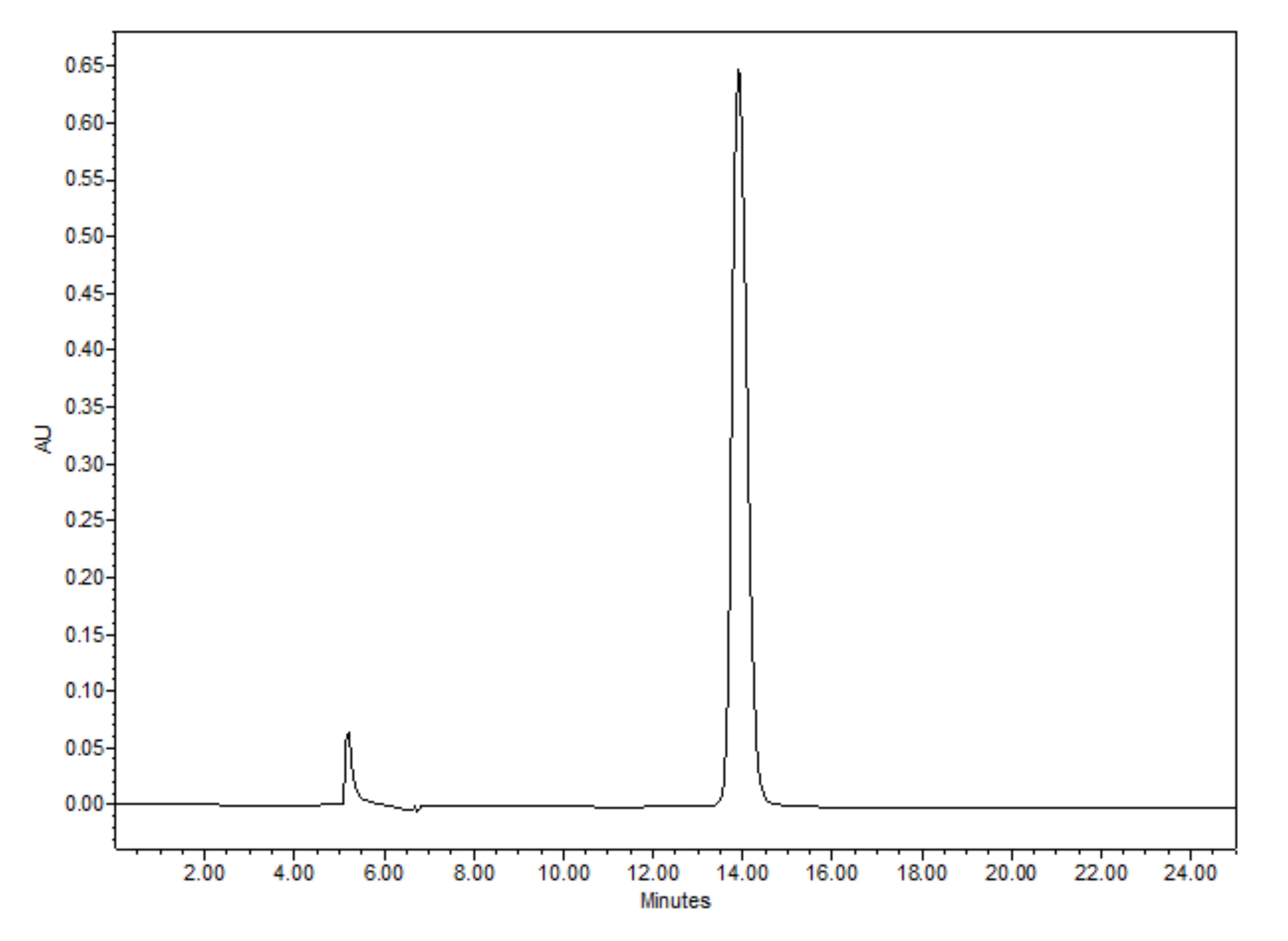

Supplement: Figure S1 — HPLC experimental conditions were: Instrument: Waters 515 with auto-sampler and the photo diode array (PDA) detector (Waters 2996); HPLC column: Waters spherisorb symmetry (C18 (5.0 μm) 250 mm × 4.6 mm); Mobile phases: 0.1% trifluoroacetic acid (TFA): acetonitrile (85: 15, v/v); Flow rate: 1.0 ml/min; Cycle time of analysis was 25 min at 30°C. (TIF) [file pone.0068710.s001.tif]

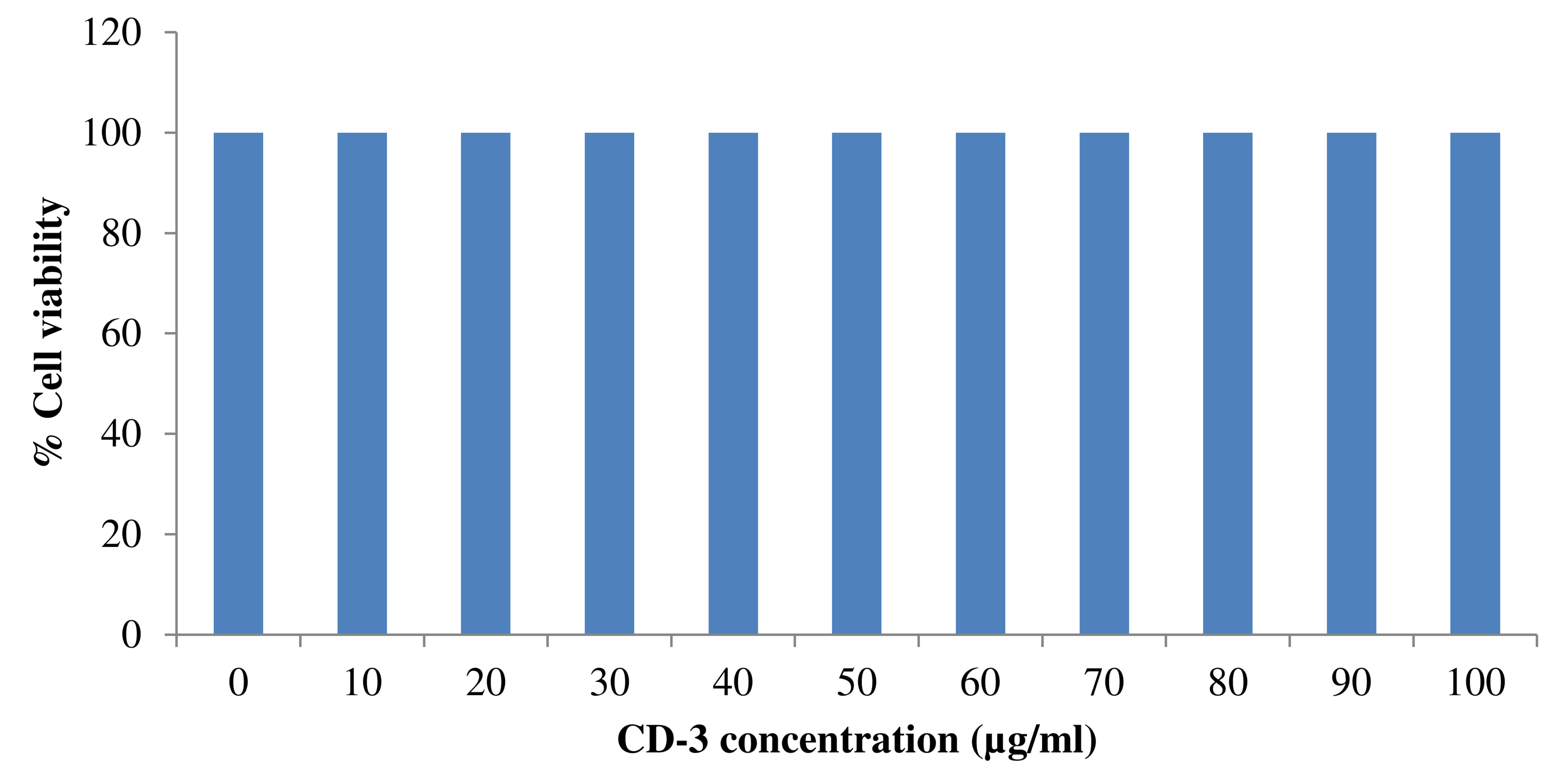

Supplement: Figure S2 — (TIF) [file pone.0068710.s002.tif]
