# Supplementary material for: Growth Inhibition and Apoptosis Induction by (+)-Cyanidan-3-ol in Hepatocellular Carcinoma
Source: PLoS One. 2013 Jul 24;8(7):e68710. doi: 10.1371/journal.pone.0068710 (PMC3722203; doi:10.1371/journal.pone.0068710)
Supplement: Table S1 — (DOCX) [file pone.0068710.s003.docx]

**Table S1 The oligonucleotide primer pairs used in reverse transcription polymerase chain reaction.**

| **Genes** | | | **Primers (5ʹ-3ʹ)** | **Annealing temperature (°C)** |
| --- | --- | --- | --- | --- |
| **Human PCR primers** | | | | |
| p53 | F | | GTTCCGAGAGCTGAATGAGG | 55 |
|  | R | | TTATGGCGGGAGGTAGACTG |  |
| mdm2 | F | | ATGGCCTGCTTACATGTGC | 56 |
|  | R | | TGAATTGAGGCATTTTCTCAC |  |
| p65 | F | | TCATCTTCCCGGCAGAGCCAG | 56 |
|  | R | | GTGGGTCTTGGTATCTGT |  |
| c-jun | F | | ATGGGCACATCACCACTACA | 57 |
|  | R | | TGAGTTGGCACCCACTGTTA |  |
| c-fos | F | | TAGTTAGTAGCATGTTGAGCCAGG | 59 |
|  | R | | ACCACCTCAACAATGCATGA |  |
| Bax | F | | CAGCTGCACCTGACG | 58 |
|  | R | | ATGCACCTACCCAGC |  |
| Bcl-2 | F | | ATGGACGGGTCCGGGGAG | 60 |
|  | R | | TCAGCCCATCTTCTTCCA |  |
| Cytochrome-c | F | | TTTGGATCCAATGGGTGATGTTGAG | 60 |
|  | R | | TTTGAATTCCTCATTAGTAGCTTTTTTGAG |  |
| Caspase-3 | F | | CAGTGGAGGCCGACTTCTTG | 54 |
|  | R | | TGGCACAAAGCGACTGGAT |  |
| Caspase-7 | F | | AGTGACAGGTATGGGCGTTC | 57 |
|  | R | | CGGCATTTGTATGGTCCTCT |  |
| Caspase-8 | F | | GGGAAGTGTTTTCACAGGTT | 58 |
|  | R | | TTCTTGCTTCCTTTGCGGAAT |  |
| Caspase 9 | F | | CTGCGAACTAACAGGCAAGC | 57 |
|  | R | | CTAGATATGGCGTCCAGCTG |  |
| β-actin | F | | AATCTGGCACCACACCTTCTAC | 54 |
|  | R | | CATCTTCCACACCACGGTCTAA |  |
| **Mouse PCR primers** | | | | |
| p53 (Exon 4-7) Primer A | | F | GGGACAGCCAAGTCTGTTATG | 54 |
|  | | R | GGAGTCTTCCAGTGTGATGAT |  |
| p53 (Exon 10-11) Primer B | | F | GTTCCGGGAGCTGAATGAGG | 55 |
|  | | R | TTATGGCGGGAGGTAGACTG |  |
| Mdm2 | | F | CCAGGCCAATGTGCAATACC | 57 |
|  | | R | AAGATGCTGGACCCTTAGTG |  |
| p65 | | F | TGGCGAGAGAAGCACAGATA | 57 |
|  | | R | TGTTGGTCTGGATTCGCTG |  |
| c-jun | | F | ATGGGCACATCACCACTACA | 57 |
|  | | R | TGAGTTGGCACCCACTGTTA |  |
| c-fos | | F | TTCCTGGCAATAGCGTGTTC | 56 |
|  | | R | TTCAGACCACCTCGACAATG |  |
| Bax | | F | GGATGCGTCCACCAAGAAGC | 58 |
|  | | R | GGAGGAAGTCCAGTGTCCAGCC |  |
| Bcl-2 | | F | GAGACA GCC AGG AGA AAT CA | 55 |
|  | | R | CCTGTG GAT GAC TGA GTA CC |  |
| Cytochrome-c | | F | ACTTGTTTCCAGATTGTCCTC | 58 |
|  | | R | GCTAAAGCGCATGCTCCAGACTG |  |
| Caspase-3 | | F | TGTCATCTCGCTCTGGTACG | 57 |
|  | | R | AAATGACCCCTTCATCACCA |  |
| Caspase-7 | | F | CAGACCGCTCCTCTATCATCT | 55 |
|  | | R | CATCGGTCTCCCCTAAAATG |  |
| Caspase-8 | | F | GGCATCTGCTTTCCCTTGTTC | 59 |
|  | | R | ATCTTACGACGACTGCACTGC |  |
| Caspase 9 | | F | ATGACCACCACAAAGCAGTCC | 58 |
|  | | R | CGTGACCATTTTCTTGGCAG |  |
| β-actin | | F | ATCCGTAAAGACCTCTATGC | 55 |
|  | | R | AACGCAGCTCAGTAACAGTC |  |

F, forward; R, reverse.
